# Supplementary material for: The spatiotemporal scaling laws of urban population dynamics
Source: Nat Commun. 2025 Mar 24;16:2881. doi: 10.1038/s41467-025-58286-4 (PMC11933343; doi:10.1038/s41467-025-58286-4)
Supplement: Supplementary file 1 — Supplementary Information [file 41467_2025_58286_MOESM1_ESM.pdf]

Supplementary Information

The spatiotemporal scaling laws of urban population fluctuations

Xingye Tan, Bo Huang*, Michael Batty, Weiyu Li, Qi Ryan Wang, Yulun Zhou, and Peng Gong

*Corresponding author: Bo Huang (bohuang@hku.hk)

Table of Contents:

Supplementary Note 1 (Study areas): 2

Supplementary Note 2 (Urban spatial structure and centrality): 4

Supplementary Note 3 (Sensitivity analysis): 10

Supplementary Note 4 (Fractal time series): 19

Supplementary Note 5 (Identified locations of urban core functional areas or hot spots): 22

Supplementary Tables: 30

Supplementary Figures: 39

References..45

Supplementary Note 1 (Study areas):

Six representative study cases from around the world are selected in this study, including four Chinese cities (Beijing, Shanghai, Guangzhou, and Shenzhen), one European city (Milan in Italy), and one North American metropolitan area (Greater Boston in the United States). In addition to their apparent distinctions in their physical geographical location, urban development history, and culture, they also show diversity in spatial range, population size, function and status, urban centrality, and urban spatial structure. Each is described briefly below.

Beijing, the capital of China, is an international city and a national political, cultural, and scientific and technological hub. In 2018, the city governed 16 districts under its jurisdiction, covering a municipal area of approximately 16,800 km^2 with a permanent population of about 21.54 million.

Shanghai is a municipality directly under the central government of China, serving as a provincial administrative unit. It plays a vital role in promoting China's economic development as a national center of international trade and economic exchange, shipping, and scientific and technological innovation. The city encompasses 16 districts, spanning a total area of approximately 6,340.5 km^2. In 2018, the city's population was about 24.24 million.

Guangzhou, the capital of Guangdong province, is recognized as a National Central City, an international trade center, and a comprehensive transportation hub. In 2018, the city encompassed 11 districts, covering a total area of nearly 7,434.4 km^2 with a population of approximately 9.28 million.

Shenzhen is a sub-provincial city in Guangdong province. It is designated as a special economic zone, a national economic center, and an international city, sharing a border with Hong Kong. The city comprised nine districts, spanning a total area of approximately 1,997.5 km^2 with a permanent population of nearly 13.03 million in 2018.

Milan, the capital of the Lombardy region and the second-most populous city in Italy after Rome, is regarded as the national industrial and financial center. It is also an alpha global city and recognized as one of the four global fashion capitals. Administratively, Milan is divided into nine

zones, covering an area of about 182.7 km² and had a population of nearly 1.32 million in 2013.

Greater Boston in this study roughly refers to the Boston–Worcester–Providence CSA (Combined Statistical Area) located in the Northeast United States. This broad region includes the core city of Boston, the capital and largest city of Massachusetts, along with surrounding cities and towns. Renowned as a center for innovation, higher education, and historical significance, it covers a total area of about 23,800 km² and had a population of approximately 8.4 million in 2020, ranking as the sixth most populous CSA in the U.S.

Readers need to note that these definitions of cities are not standardised but are the best for enabling comparative work at the present time. The problems and pitfalls of defining cities for comparative work such as this are outlined in Dong *et al*.[1]

Supplementary Note 2 (Urban spatial structure and centrality):

According to the Beijing Urban Master Plan (2016–2035), Beijing's urban spatial system is divided into five main levels, as shown in Fig. S1 (left). The first level is the functional core area of the capital, consisting of the Xicheng and Dongcheng districts. The second level comprises the central urban area, including the urban core and the surrounding Chaoyang, Haidian, Fengtai, and Shijingshan districts. The third level features Tongzhou New Town as the city sub-center, responsible for municipal administrative functions. The fourth level includes the new towns of Shunyi, Daxing, Yizhuang, Changping, and Fangshan, located on the plains, serving peripheral areas and residents outside the central urban area. Finally, the mountainous districts of Mentougou, Pinggu, Huairou, Miyun, and Yanqing are primarily responsible for ecological conservation to ensure sustainable urban development.

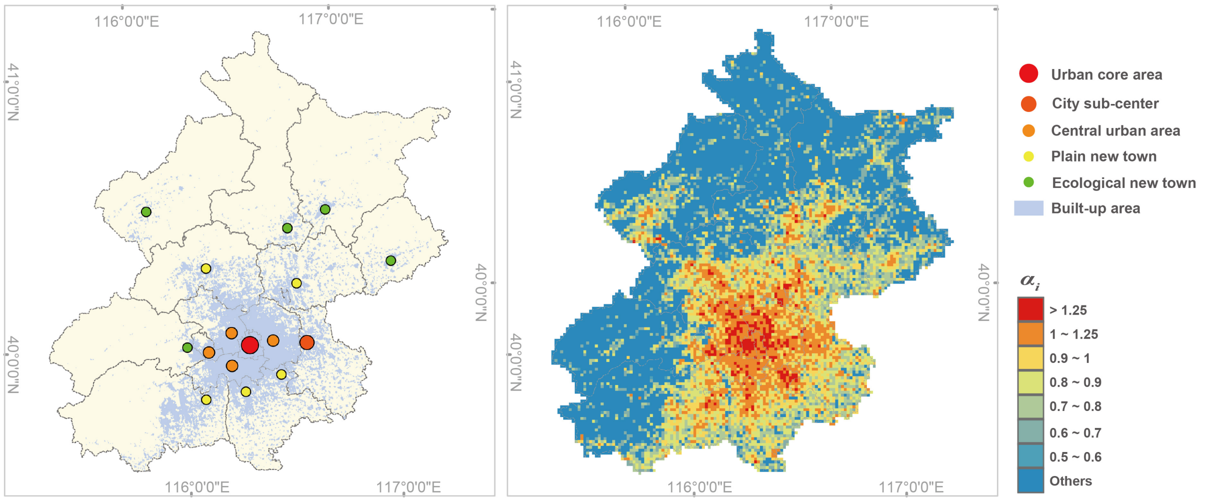

Fig. S1 | Comparison between urban structure (left) and spatial patterns of temporal scaling exponents α_i (right) of Beijing. [Note: This figure is Powered by Esri. The built-up areas shown on the left-side map were derived from the Land Cover-Climate Change Initiative data[2] provided by the European Space Agency (© 2017 ESA Climate Change Initiative - Land Cover led by UCLouvain) for 2018, corresponding to when the mobile data were extracted for Beijing. The image on the right shows the same spatial arrangement of the classified α_i as in Fig. 4.]

The Shanghai Urban Master Plan (2016–2035) outlines the construction of a three-level urban system for Shanghai, as shown in Fig. S2 (left). This includes a main urban area, new towns, and new counties. The main urban area, which plays a core role in strengthening Shanghai's urban functionality and demonstrating the quality of its urban space, includes the city center (People's Square) and the surrounding main urban areas within the outer-ring expressway. It covers Huangpu, Jing'an, Hongkou, Yangpu, most of Xuhui and Putuo, and parts of Changning, Minhang, Baoshan,

and Pudong. Peripheral support nodes like Hongqiao, Chuansha, Baoshan, and Minhang, complement the core urban functions together with the central city. The new towns, including Jiading, Songjiang, Qingpu, Fengxian, and Nanhui, are expected to become developmental nodes in the Yangtze River Delta. The new counties primarily serve the surrounding rural areas.

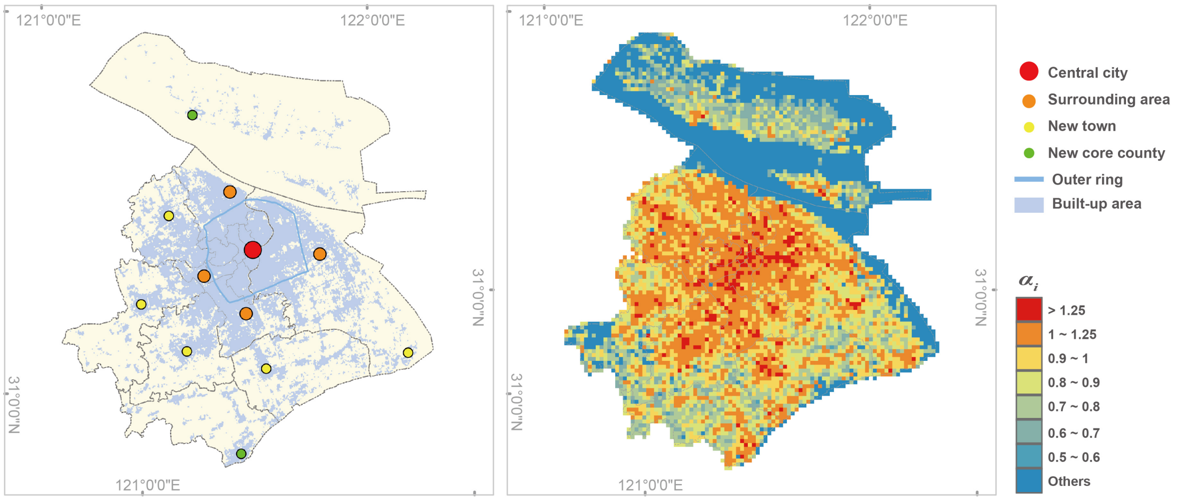

Fig. S2 | Comparison between urban structure (left) and spatial patterns of temporal scaling exponents α_i (right) of Shanghai. [Note: This figure is Powered by Esri. The built-up areas shown on the left-side map were derived from the Land Cover-Climate Change Initiative data[2] provided by the European Space Agency (© 2017 ESA Climate Change Initiative - Land Cover led by UCLouvain) for 2018, corresponding to when the mobile data were extracted for Shanghai. The image on the right shows the same spatial arrangement of the classified α_i as in Fig. 4.]

Based on the Guangzhou Urban Master Plan (2010–2020), Guangzhou's urban spatial structure is organized as a multi-center network comprising one metropolitan area, two new towns, and three city sub-centers, as depicted in Fig. S3 (left). The metropolitan area, central to Guangzhou, encompasses the Yuexiu, Haizhu, Liwan, and Tianhe districts, along with parts of the Huangpu, Baiyun, and Panyu districts. It is designated to perform National Central City functions, leading the development of high-end urban core services. The two new towns – the coastal town of Nansha and the eastern town of the former Luogang, now part of Huangpu District – are strategic areas leading the transformation outlined in the plan by improving service functions, fostering coordinated development with industries, and accelerating population agglomeration. Huadu, Conghua, and Zengcheng, the three city sub-centers, promote the overall development of Guangzhou's urban and rural areas and provide services to the population beyond the metropolitan area.

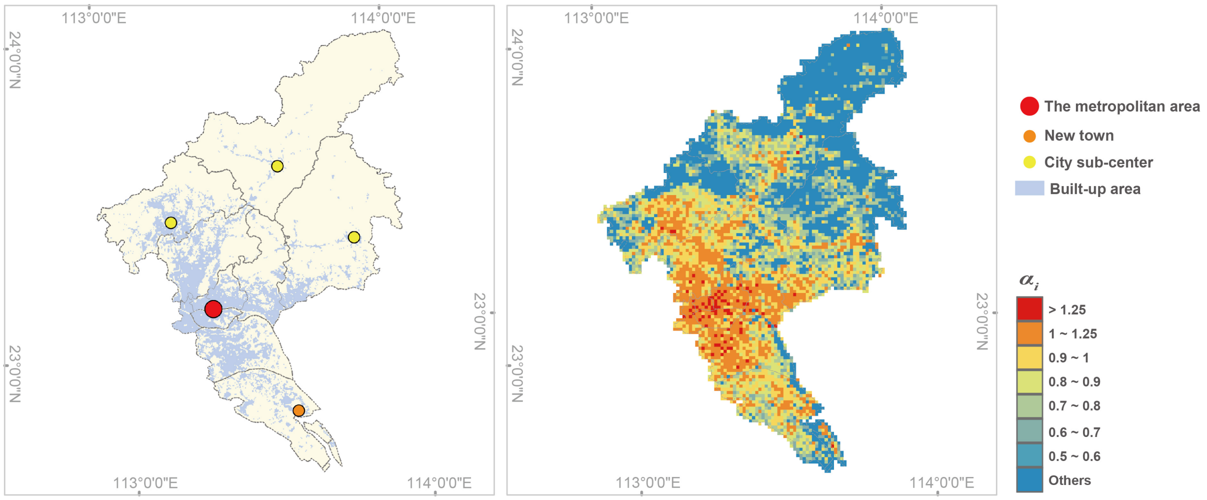

Fig. S3 | Comparison between urban structure (left) and spatial patterns of temporal scaling exponents α_i (right) of Guangzhou. [Note: This figure is Powered by Esri. The built-up areas shown on the left-side map were derived from the Land Cover-Climate Change Initiative data[2] provided by the European Space Agency (© 2017 ESA Climate Change Initiative - Land Cover led by UCLouvain) for 2018, corresponding to when the mobile data were extracted for Guangzhou. The image on the right shows the same spatial arrangement of the classified α_i as in Fig. 4.]

As shown in Fig. S4 (left), the Shenzhen City Master Plan (2010–2020) aimed to establish a three-level urban spatial center system for Shenzhen, comprising two main city centers, five city sub-centers, and eight city cluster centers. The two main city centers, Futian-Luohu and Nanshan-Qianhai, provide high-end regional services and form a dual-center structure with a regional radiation function. The five urban sub-centers – Longgang, Longhua, Guangming, Pingshan, and Yantian – are responsible for providing comprehensive services and carrying out municipal functions in their respective districts. Additionally, eight city cluster centers, namely Aviation City, Shajing, Songgang, Guanlan, Pinghu, Buji, Henggang, and Kuichong, serve as secondary integrated service centers within their districts.

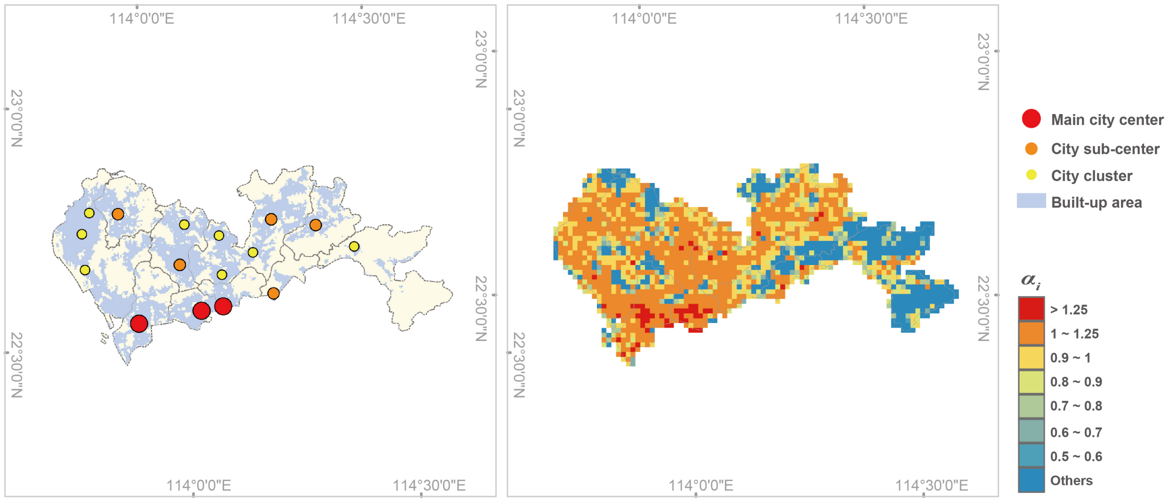

Fig. S4 | Comparison between urban structure (left) and spatial patterns of temporal scaling exponents α_i (right) of Shenzhen. [Note: This figure is Powered by Esri. The built-up areas shown on the left-side map were derived from the Land Cover-Climate Change Initiative data[2] provided by the European Space Agency (© 2017 ESA Climate Change Initiative - Land Cover led by UCLouvain) for 2018, corresponding to when the mobile data were extracted for Shenzhen. The image on the right shows the same spatial arrangement of the classified α_i as in Fig. 4.]

According to the latest Territorial Government Plan (PGT) of Milan (2030), the city spatially develops along radial axes and concentric circles (e.g., Navigli circle, city wall circle, tram loop, railway strip). Over recent decades, it initially underwent a "leapfrog" development within metropolitan and regional boundaries, gradually merging and connecting to form the existing settlement framework. Its previous PGT (2012) proposed the development concept of a multicentric metropolitan network between the city center and suburbs. This concept promotes the rebalancing of functions between the center and periphery and is conducive to intercity projects, aiming to create a more balanced city. The main transportation networks and conceptual planning centers of Milan are shown in Fig. S5 (left).

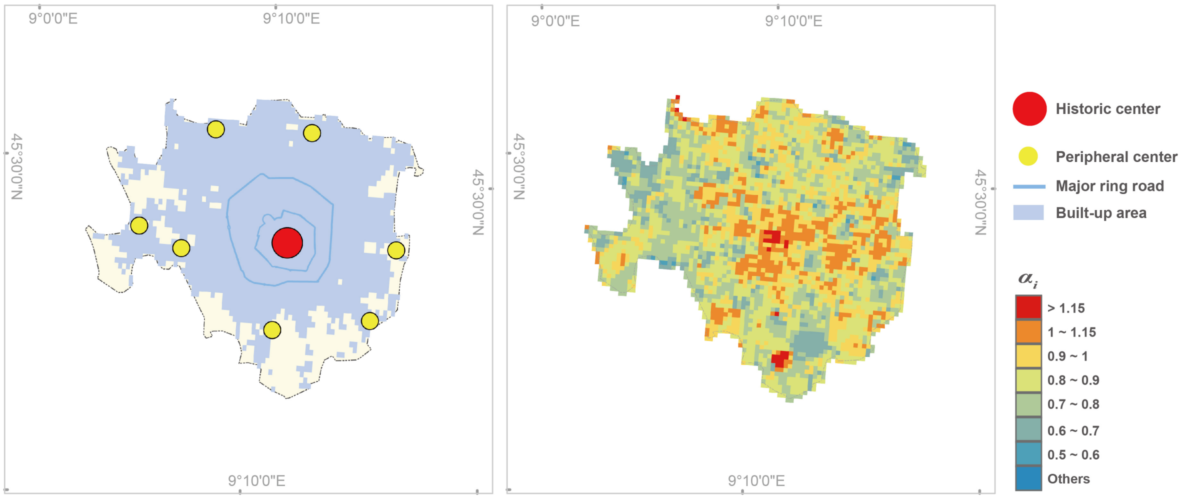

Fig. S5 | Comparison between urban structure (left) and spatial patterns of temporal scaling exponents α_i (right) of Milan. [Note: This figure is Powered by Esri. The built-up areas shown on the left-side map were derived from the Land Cover-Climate Change Initiative data[2] provided by the European Space Agency (© 2017 ESA Climate Change Initiative - Land Cover led by UCLouvain) for 2013, corresponding to when the mobile data were extracted for Milan. The image on the right shows the same spatial arrangement of the classified α_i as in Fig. 4.]

The regional plan for Greater Boston, MetroFuture (2030), defines three main types of development zones in the metropolitan area: the metropolitan core, regional hubs, and suburban centers (the left-side map in Fig. S6). The metropolitan core, mainly covering the high-density cities of Boston, Cambridge, Somerville, Revere, Everett, and Chelsea, serves as the anchor of the regional economy. The regional hubs, which include urban-scale downtown centers outside of the core such as the clusters of Woburn-Burlington, Peabody-Beverly, and Framingham-Natick, as well as the cities of Quincy, Waltham, Gloucester, Marlborough, and Lynn, and the towns of Norwood and Milford, are considered potential areas for industry and downtown rejuvenation. The suburban centers are major town centers planned to expand housing choices and increase tax revenue in the future. Additionally, important nodal cities beyond the planned scope but covered by our data, such as Worcester, Providence, Manchester, Nashua, Lowell, Portsmouth, Fall River, and New Bedford, are also marked in Fig. S6 (left).

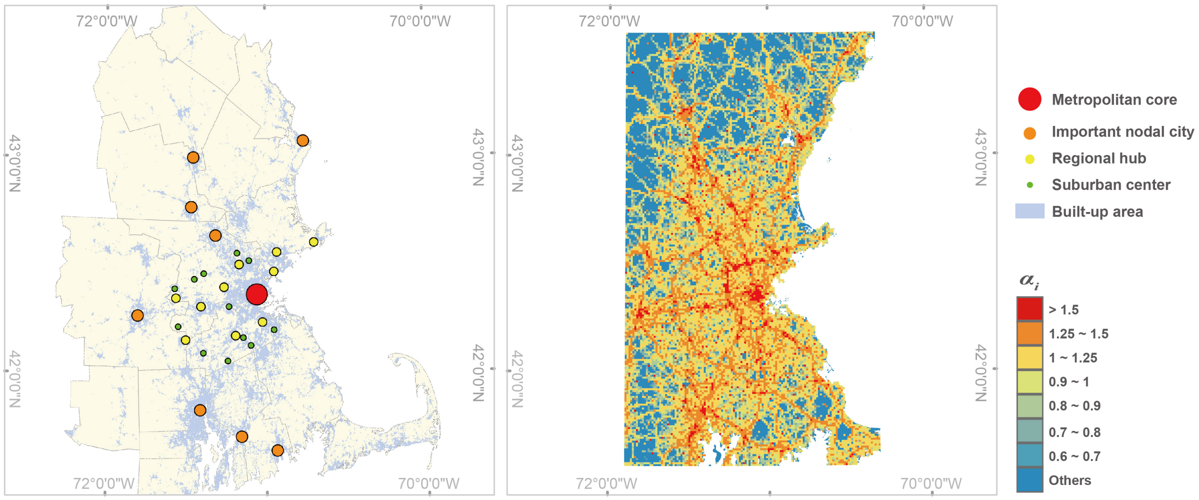

Fig. S6 | Comparison between urban structure (left) and spatial patterns of temporal scaling exponents α_i (right) of Greater Boston. [Note: This figure is Powered by Esri. The built-up areas shown on the left-side map were derived from the Land Cover-Climate Change Initiative data[2] provided by the European Space Agency (© 2017 ESA Climate Change Initiative - Land Cover led by UCLouvain) for 2020, corresponding to when the mobile data were extracted for Greater Boston. The image on the right shows the same spatial arrangement of the classified α_i as in Fig. 4.]

Supplementary Note 3 (Sensitivity analysis):

The main purpose of the sensitivity analysis here is to address any occasional biases potentially caused by our experimental design for ensuring the generality and objectivity of our findings and the feasibility of our proposed models. These potential biases may include the effects of annular segmentation of grid cells and the impact of spatial distance sampling on analytical results. Therefore, we modified the concentric ring structure used in the main text by spatially translating these rings while keeping the same centers and annular width r_c in each city. This alteration introduced different situations of cell segmentation and generated new sets of radius sequences different from those in Table S3, allowing us to examine the above issues. To avoid the overlap of annular structures, the translation value should be less than the width of the ring (For Milan, the translation distance accuracy is 0.01 km, while for the other five cases, it is 0.1 km). We generated two sets of translation values using the method of uniformly distributed pseudorandom integers in MATLAB. Considering that this random method may result in two adjacent translations or translations adjacent to the original structure, two additional sets of translations with certain spacing were artificially determined. We then measured corresponding parameters and indicators such as the mean population fluctuation, $F(s,r)$, and the urban density of population and points of interest (POI), $\rho(r)$, for examination.

We re-examined the temporal (see Fig. S7) and spatial (see Fig. S8) scaling relations of urban population fluctuations with these four new sets of measurement results for all six focal cases. Table S1 summarizes the information about the linear goodness of fit for these scaling relations. The approximate linearity of the plots in either Fig. 3 or Figs. S7, S8 confirms that the spatiotemporal scaling laws of urban population fluctuations that we previously revealed are almost unaffected by our experimental design and measurement methods, thus objectively existing.

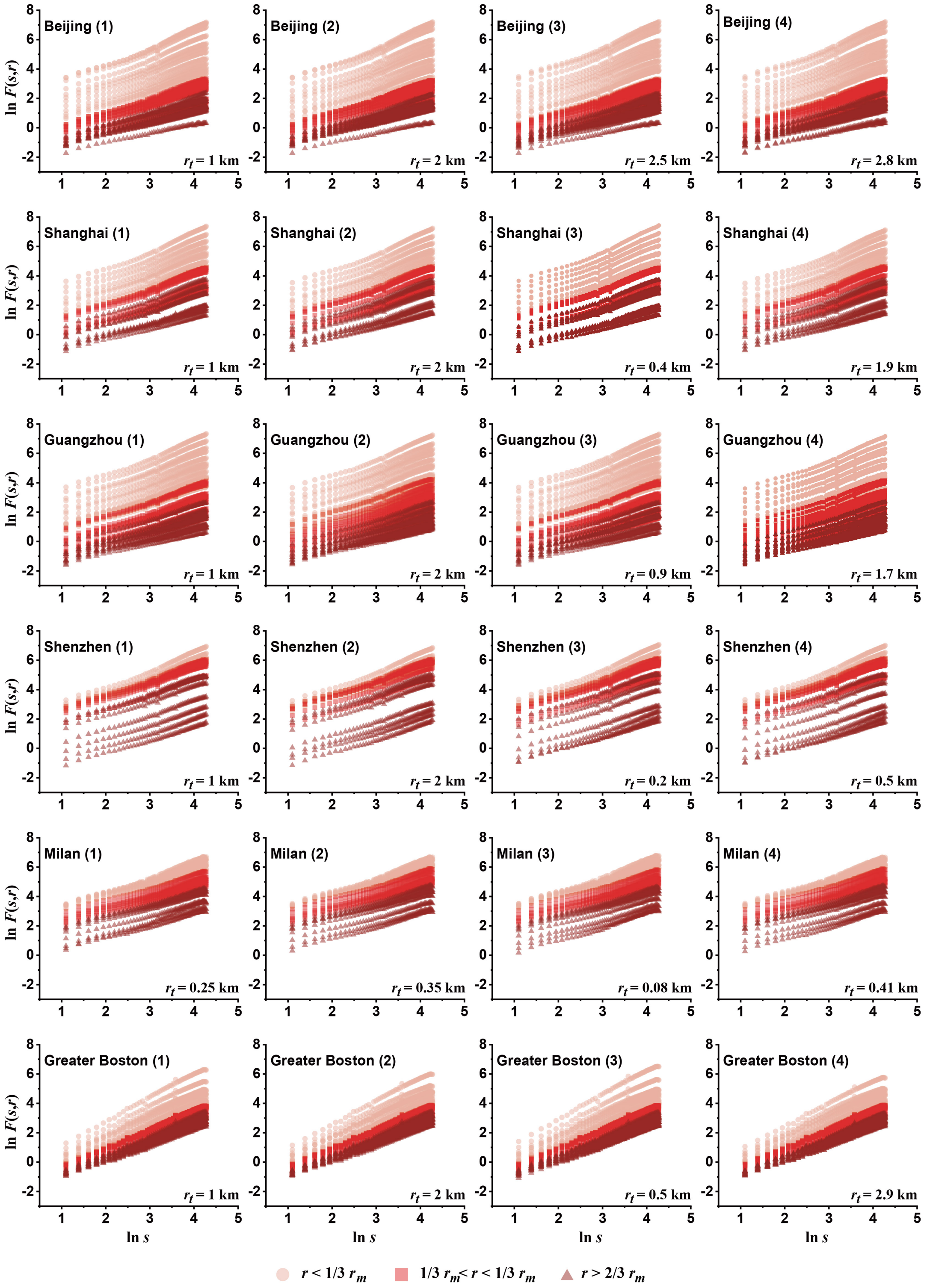

Fig. S7| Temporal scaling relations of urban population fluctuations of four new experimental groups (1-4) across the six focal cases. r_t refers to translation distance. The translation distances of groups 1-2 were artificially determined, while those of groups 3-4 were randomly generated. r_m refers to the maximum radius from the closest main urban center for each case.

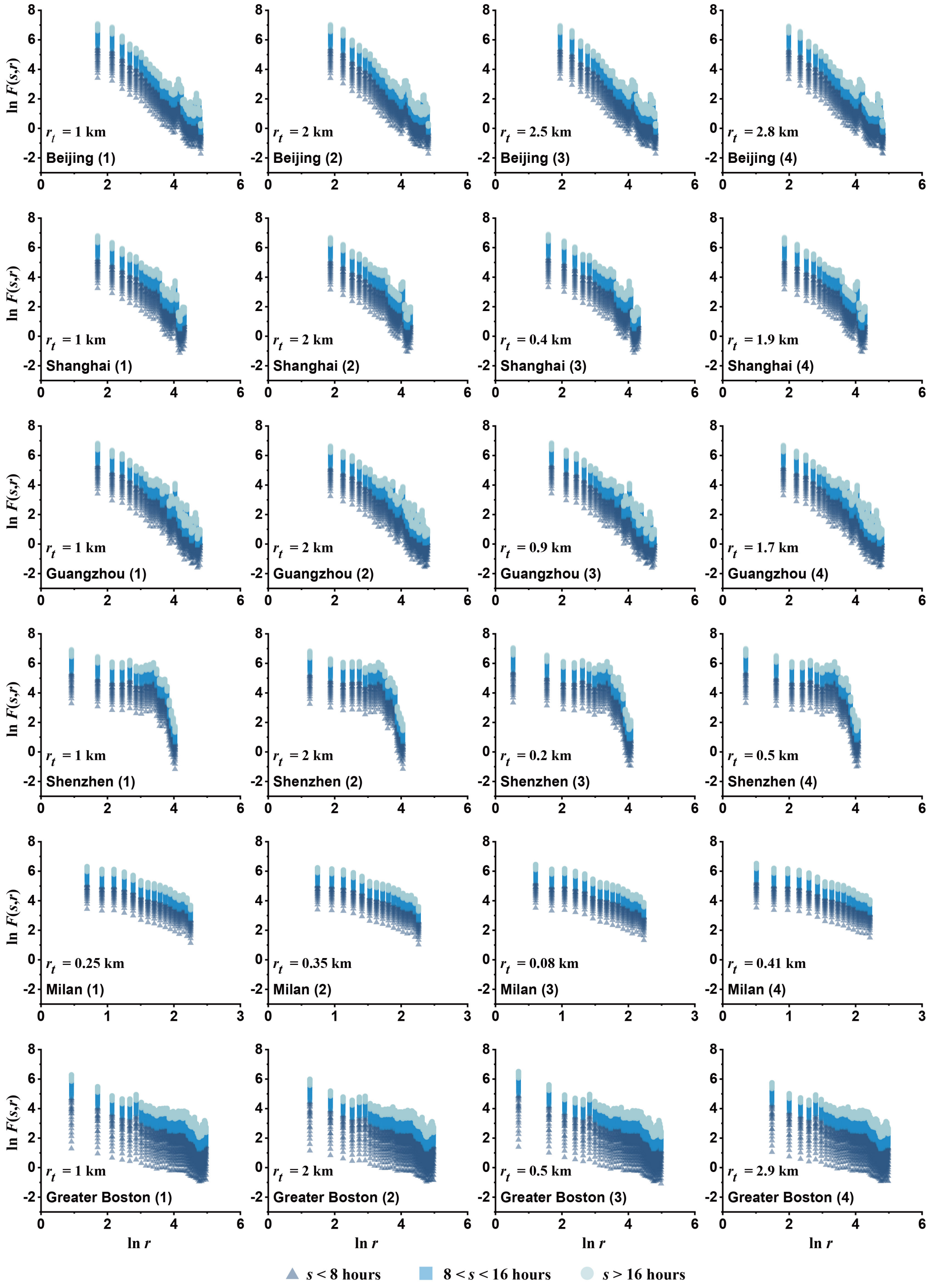

Fig. S8| Spatial scaling relations of urban population fluctuations of four new experimental groups (1-4) across the six focal cases. r_t refers to translation distance. The translation distances of groups 1-2 were artificially determined, while those of groups 3-4 were randomly generated.

Table S1| Ranges and averages of the linear goodness of fit, R^2, for temporal and spatial scaling relations of urban population fluctuations of the original group (0) and the four new experimental groups (1-4).

City Name	ln $F(s,r)$ vs. ln s		ln $F(s,r)$ vs. ln r	
	R^2	Avg. R^2	R^2	Avg. R^2
Beijing (0)	0.973 – 0.999	0.994	0.943 – 0.963	0.947
Beijing (1)	0.970 – 0.999	0.994	0.945 – 0.964	0.949
Beijing (2)	0.984 – 0.998	0.994	0.945 – 0.963	0.949
Beijing (3)	0.976 – 0.999	0.994	0.946 – 0.963	0.949
Beijing (4)	0.974 – 0.999	0.994	0.944 – 0.963	0.948
Shanghai (0)	0.985 – 0.998	0.992	0.888 – 0.923	0.900
Shanghai (1)	0.984 – 0.998	0.991	0.895 – 0.927	0.906
Shanghai (2)	0.986 – 0.998	0.992	0.892 – 0.925	0.903
Shanghai (3)	0.985 – 0.998	0.992	0.890 – 0.923	0.900
Shanghai (4)	0.985 – 0.998	0.992	0.893 – 0.925	0.903
Guangzhou (0)	0.985 – 0.998	0.994	0.918 – 0.945	0.918
Guangzhou (1)	0.987 – 0.998	0.994	0.924 – 0.947	0.931
Guangzhou (2)	0.987 – 0.999	0.994	0.928 – 0.950	0.936
Guangzhou (3)	0.987 – 0.998	0.994	0.924 – 0.947	0.932
Guangzhou (4)	0.988 – 0.999	0.994	0.926 – 0.949	0.934
Shenzhen (0)	0.988 – 0.997	0.992	0.834 – 0.922	0.893
Shenzhen (1)	0.987 – 0.996	0.992	0.780 – 0.902	0.865
Shenzhen (2)	0.987 – 0.995	0.992	0.773 – 0.917	0.870
Shenzhen (3)	0.987 – 0.997	0.992	0.818 – 0.930	0.898
Shenzhen (4)	0.987 – 0.997	0.992	0.800 – 0.931	0.893
Milan (0)	0.988 – 0.996	0.992	0.943 – 0.961	0.956
Milan (1)	0.986 – 0.995	0.992	0.929 – 0.947	0.941
Milan (2)	0.989 – 0.995	0.992	0.924 – 0.942	0.936
Milan (3)	0.988 – 0.996	0.992	0.939 – 0.958	0.952
Milan (4)	0.989 – 0.996	0.992	0.942 – 0.962	0.956
Greater Boston (0)	0.994 – 0.998	0.997	0.915 – 0.944	0.926
Greater Boston (1)	0.993 – 0.998	0.997	0.900 – 0.937	0.914
Greater Boston (2)	0.994 – 0.998	0.997	0.892 – 0.935	0.908
Greater Boston (3)	0.993 – 0.998	0.997	0.907 – 0.939	0.920
Greater Boston (4)	0.994 – 0.998	0.997	0.883 – 0.932	0.902

[Note: Avg. R^2 refers to the average of the goodness of fit, R^2.]

To test the reliability of our model and parameters, we further used the estimated model parameters (listed in Table 3) to predict the mean fluctuation, $F(s,r)$, corresponding to four sets of radii at varying temporal scales. We then compared all the predicted results with the actual measured values on a logarithmic scale. The comparison results, shown in Fig. S9, indicate that our model and parameters are still valid for cases that were not used for parameter estimation.

Finally, we verified the allometric relations between population fluctuations and urban densities of population (Fig. S10) and POI (Fig. S11) with the four groups of new measurement results. This was to ensure that our explanation of the spatiotemporal patterns of urban population dynamics remains rational and reliable. The summary results of the linear goodness of fit for the double-logarithmic relationships between population fluctuations and urban densities, listed in Table S2, suggest that the urban allometric relations as stated in the main text still hold, even if we change the experimental design and measurement methods.

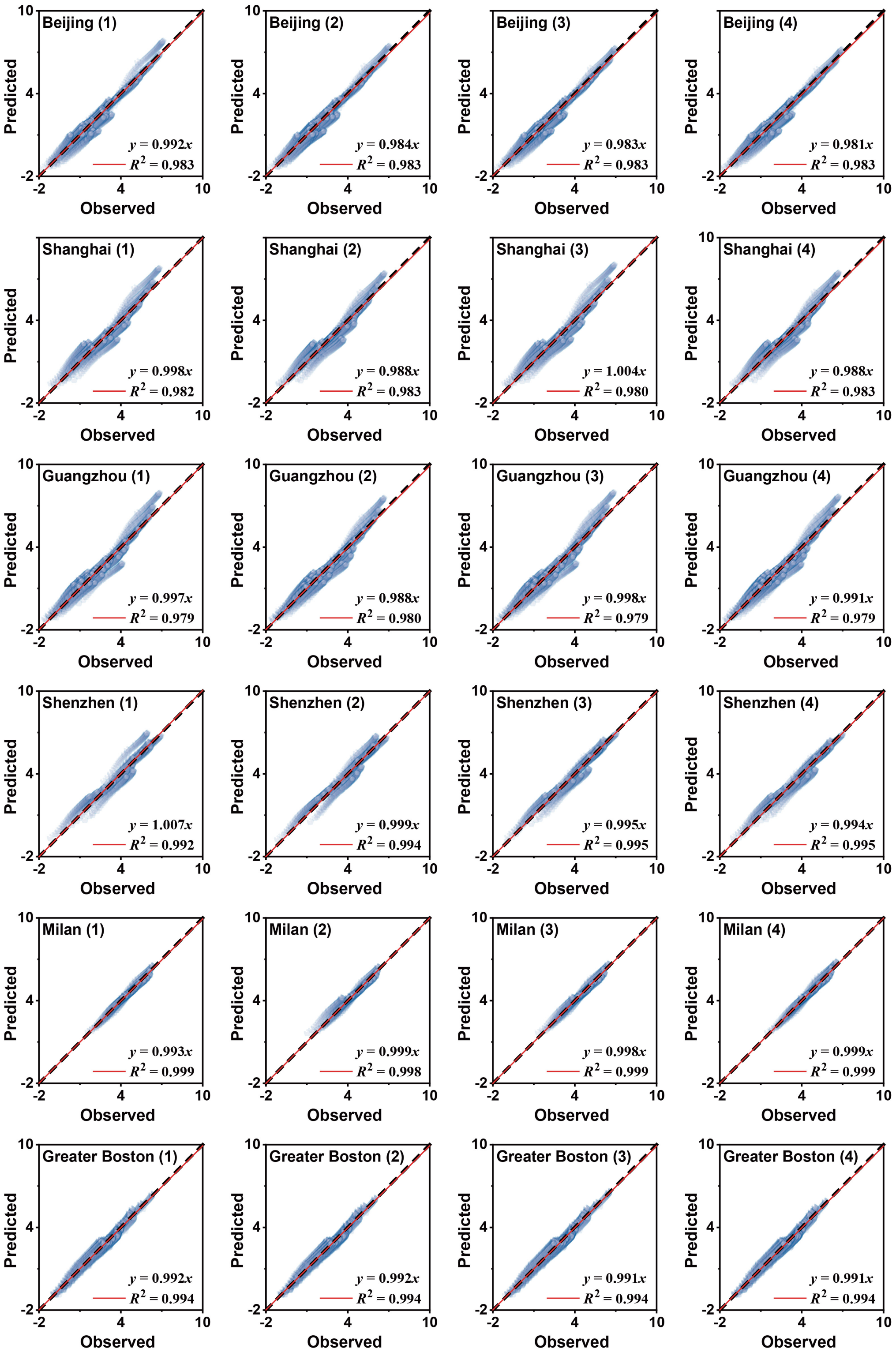

Fig. S9| Model validation of urban population fluctuations using four new experimental groups (1-4) for the six focal cases. The dashed line represents a perfect prediction of the observation (i.e., $y = x$). The closer the slope coefficient of plots (fitted by the red lines) is to 1, the better the predicted results match the observed. The logarithmic expression ensures the comparability across time and space scales within a plot by eliminating the dimensional inconsistencies. The group numbers and the corresponding translation distances are consistent with Figs. S7 and S8.

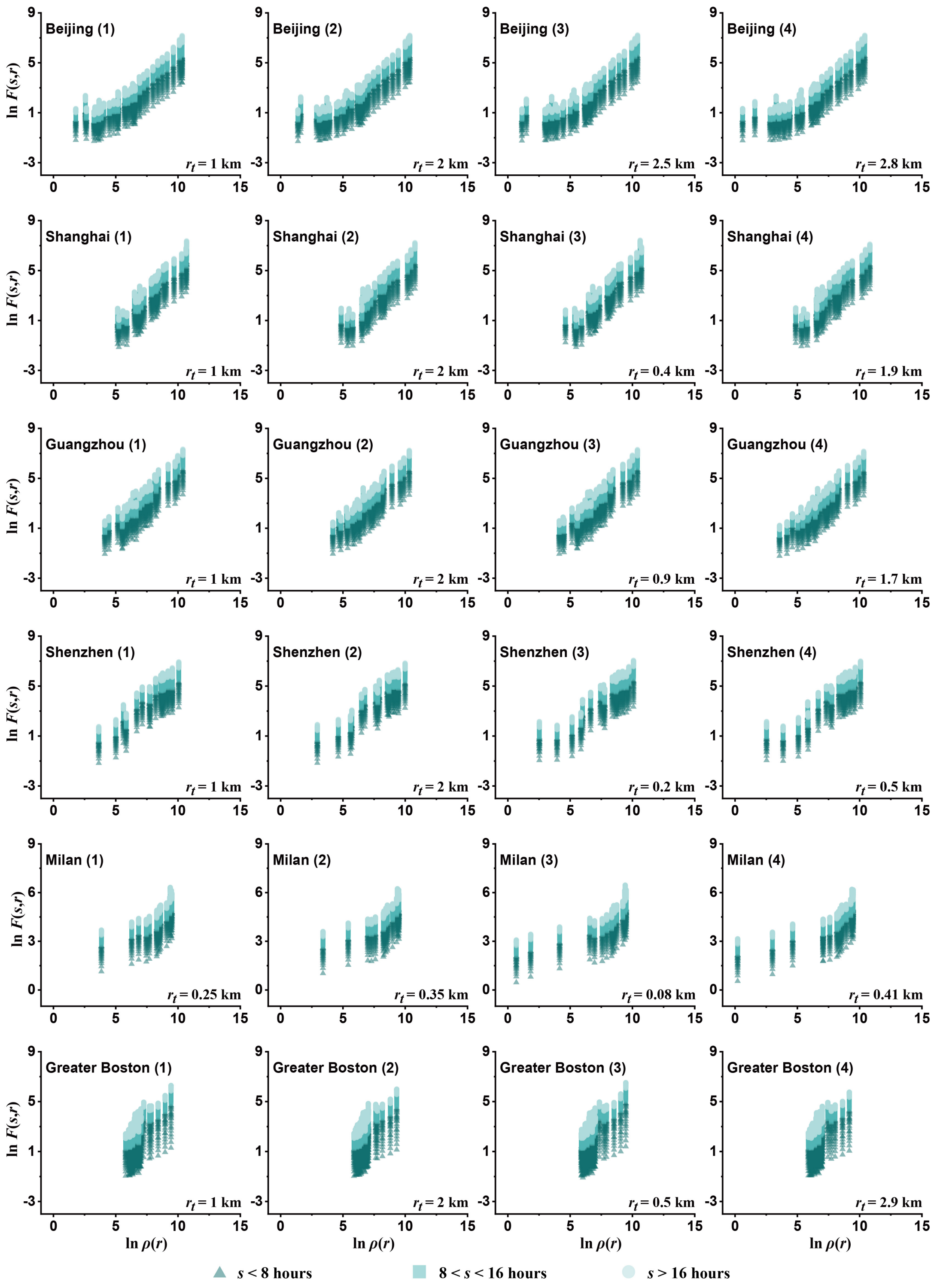

Fig. S10| Urban allometric relations between the mean population fluctuations $F(s,r)$ and population densities $\rho(r)$ of four new experimental groups (1-4) across the six focal cases. r_t refers to translation distance. The translation distances of groups 1-2 were artificially determined, while those of groups 3-4 were randomly generated.

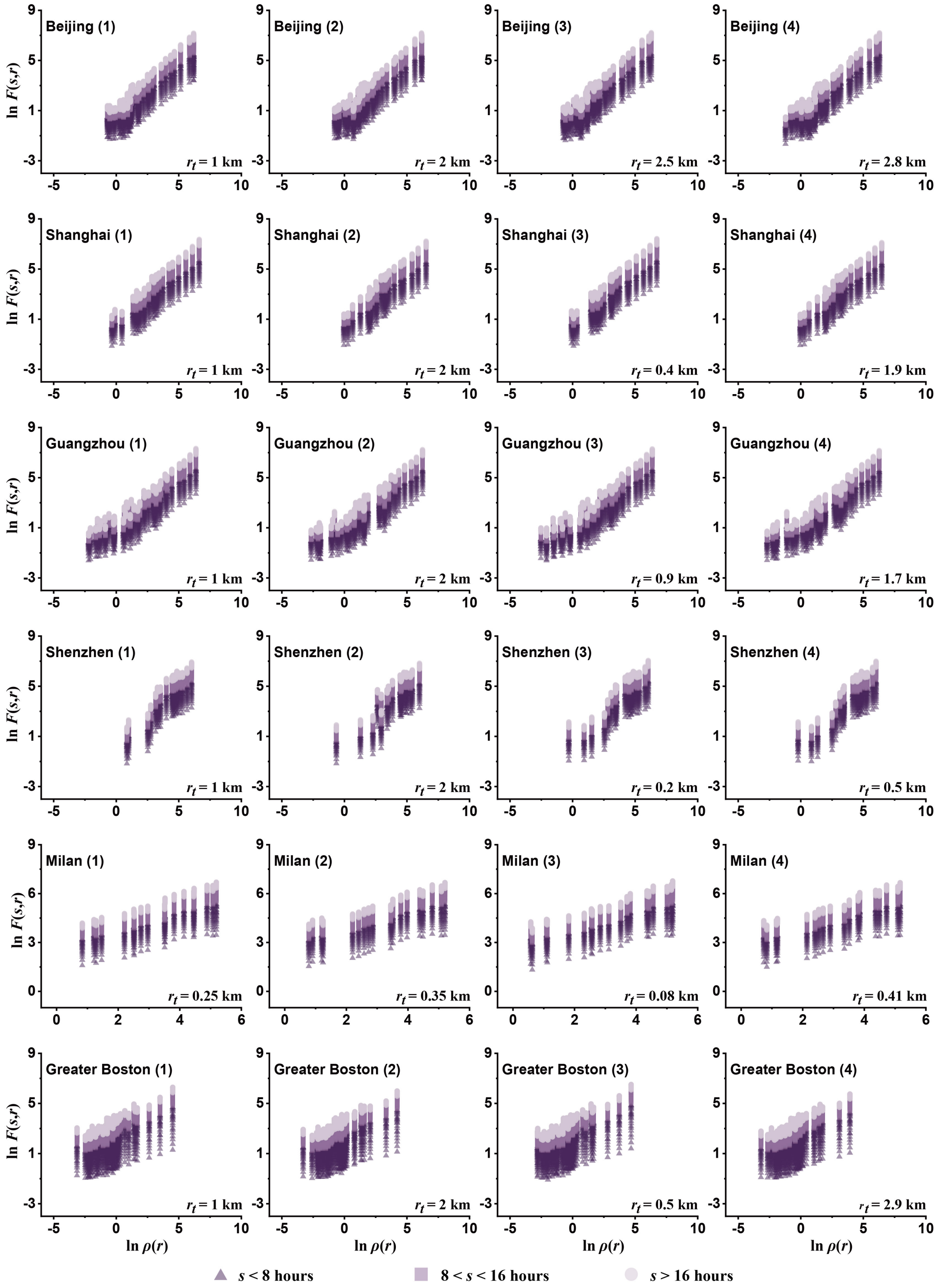

Fig. S11| Urban allometric relations between the mean population fluctuations $F(s,r)$ and POI densities $\rho(r)$ of four new experimental groups (1-4) across the six focal cases. r_t refers to translation distance. The translation distances of groups 1-2 were artificially determined, while those of groups 3-4 were randomly generated.

Table S2| Ranges and averages of the linear goodness-of-fit, R^2, for allometric relationships between urban population fluctuations and urban densities in the original group (0) and the four new experimental groups (1-4).

**City Name**	**ln $F(s,r)$ vs. ln $\rho(r)$** (Population density)		**ln $F(s,r)$ vs. ln $\rho(r)$** (POI density)	
	R^2	Avg. R^2	R^2	Avg. R^2
Beijing (0)	0.886 – 0.897	0.890	0.871 – 0.893	0.878
Beijing (1)	0.916 – 0.931	0.926	0.969 – 0.971	0.970
Beijing (2)	0.897 – 0.907	0.902	0.964 – 0.965	0.964
Beijing (3)	0.883 – 0.900	0.895	0.964 – 0.969	0.966
Beijing (4)	0.875 – 0.893	0.888	0.963 – 0.967	0.964
Shanghai (0)	0.943 – 0.958	0.948	0.876 – 0.899	0.884
Shanghai (1)	0.971 – 0.979	0.974	0.984 – 0.987	0.986
Shanghai (2)	0.957 – 0.966	0.961	0.986 – 0.989	0.987
Shanghai (3)	0.963 – 0.969	0.966	0.987 – 0.989	0.987
Shanghai (4)	0.957 – 0.968	0.962	0.985 – 0.989	0.986
Guangzhou (0)	0.914 – 0.920	0.915	0.886 – 0.898	0.892
Guangzhou (1)	0.959 – 0.967	0.963	0.948 – 0.957	0.953
Guangzhou (2)	0.962 – 0.973	0.968	0.936 – 0.952	0.946
Guangzhou (3)	0.962 – 0.970	0.966	0.943 – 0.954	0.950
Guangzhou (4)	0.959 – 0.973	0.967	0.935 – 0.949	0.944
Shenzhen (0)	0.946 – 0.966	0.956	0.905 – 0.937	0.920
Shenzhen (1)	0.951 – 0.969	0.959	0.920 – 0.949	0.934
Shenzhen (2)	0.942 – 0.956	0.950	0.865 – 0.889	0.878
Shenzhen (3)	0.938 – 0.948	0.944	0.921 – 0.936	0.930
Shenzhen (4)	0.940 – 0.949	0.945	0.928 – 0.943	0.936
Milan (0)	0.787 – 0.814	0.797	0.912 – 0.957	0.946
Milan (1)	0.788 – 0.816	0.797	0.963 – 0.982	0.977
Milan (2)	0.791 – 0.821	0.801	0.956 – 0.974	0.969
Milan (3)	0.799 – 0.834	0.812	0.957 – 0.974	0.970
Milan (4)	0.800 – 0.829	0.810	0.953 – 0.970	0.965
Greater Boston (0)	0.816 – 0.842	0.834	0.921 – 0.933	0.931
Greater Boston (1)	0.795 – 0.825	0.814	0.905 – 0.918	0.916
Greater Boston (2)	0.774 – 0.799	0.791	0.898 – 0.913	0.910
Greater Boston (3)	0.811 – 0.841	0.831	0.915 – 0.927	0.925
Greater Boston (4)	0.765 – 0.788	0.781	0.901 – 0.915	0.911

[Note: Avg. R^2 refers to the average of the goodness of fit, R^2.]

Supplementary Note 4 (Fractal time series):

When dealing with time series data from truly complex systems, it is possible to represent their periodic components by multiple characteristic frequencies or frequency bands, and the fluctuations may extend over many time scales. Consequently, no specific characteristic frequency or time scale can be observed, and the dynamics, often denoted as fractals or multifractals, are usually characterized by scaling laws[3]. Natural phenomena have demonstrated such temporally scale-free characteristics[4–6], indicating a strong interdependence between distant samples in the time domain. To investigate the scaling behavior of time series, and more importantly, to reveal the "span of interdependence" between records or their increments[7], the concept of fractals was introduced to time series analysis[7,8], thereby establishing a dimensional relationship.

Typically, for fractal time series, their sample power spectral density (PSD), $G(f)$, and frequency f follow a power law relationship: $S(f) = G(f)^2 = f^{-\gamma}$. Here, $S(f)$ is the power spectrum and γ refers to the spectral index[3,9,10]. The special case with $\gamma = 1$ is related to the famous problem of $1/f$ noise, which has been considered a sign of self-organised criticality (SOC)[11–13]. The spectral index γ can measure the correlation or persistence between adjacent values within the time series[9,14,15]. It is closely related to the Hurst exponent[16], which has been widely used to characterise long-range dependence[8,10,17]. Based on these two parameters, it is possible to classify fractal time series even further.

On the basis of the spectral index γ, which is associated with the parameter of the Hurst exponent H, fractal time series can be expressed by a dichotomous model of fractional Gaussian noise (fGn) and fractional Brownian motion (fBm)[9,10]. For one dimensional series, the critical value of γ ($-1 < \gamma < 3$) to distinguish fBm from fGn is 1. Accordingly, fractal signals with $-1 < \gamma < 1$ represent fGn and those with $1 < \gamma < 3$ represent fBm. Typically, $\gamma = 0$ indicates white noise and $\gamma = 2$ indicates Brownian motion. The temporal scaling exponent α_i ($0 < \alpha_i < 2$) has the mutual conversion relation with the spectral index γ[10], indicating that α_i is also a significant criterion to distinguish fGn and fBm with a critical value of 1[9,18]. Specifically, the fractal signals with $1 < \alpha_i < 2$ represent fBm, while those with $0 < \alpha_i < 1$ represent fGn. Particularly, when $\alpha_i = 0.5$, white noise is present. When $\alpha_i = 1.5$, ordinary Brownian motion is present[9,14,18]. $\alpha_i = 1$ (i.e., $\gamma = 1$) also

refers to the well-known phenomenon of $1/f$ noise[7,11,12,18], indicating the transitional boundary between fGn and fBm and the SOC[12,19,20].

The fundamental differences between fBm and fGn lie in their stationarity and persistence[9,15]. fGn is stationary, with its variance being a constant over time. In contrast, fBm is non-stationary, meaning that the observed variance increases with the length of the observation period, but the increments are stationary (fGn)[7,9]. Due to the difference in stationarity, compared with fGn, fBm shows stronger persistence, which is usually indicated by its increments within series[8,15,21]. Considering the statistical distinction between the two temporal processes, two sets of the Hurst exponents, H_{fBm} and H_{fGn}, are used to characterise their dependence, although they have the same value range. $H_{\mathrm{fGn}} > 0.5$ is positively correlated fGn and $H_{\mathrm{fGn}} < 0.5$ refers to negatively correlated or anti-correlated fGn. $H_{\mathrm{fBm}} > 0.5$ indicates persistent fBm and $H_{\mathrm{fBm}} < 0.5$ refers to anti-persistent fBm. $H_{\mathrm{fGn}} = 0.5$, corresponding to $\alpha_i = 0.5$, represents white noise, and $H_{\mathrm{fBm}} = 0.5$, corresponding to $\alpha_i = 1.5$, represents Brownian noise[8,9,14,22]. For both processes, the closer the Hurst exponent H is to 1, the smoother the fluctuation[9,22] (e.g., Fig. S12).

To identify fBm and fGn, the most straightforward method is to conduct PSD analysis by means of a fast Fourier transform (FFT) to estimate the spectral index γ. However, due to FFT, the estimation results commonly show noisy fluctuation superposed onto the power law spectrum, resulting in an unstable spectral index γ estimation[23]. Besides, the inherent assumption of FFT is that the signals are stationary, which limits the PSD analysis to stationary time series[3,24]. Therefore, we used the detrended fluctuation analysis to investigate and measure the temporal dynamics of population fluctuation due to its broad application to both stationary and non-stationary time series[18,25] and its ability to achieve relatively stable scaling behaviour.

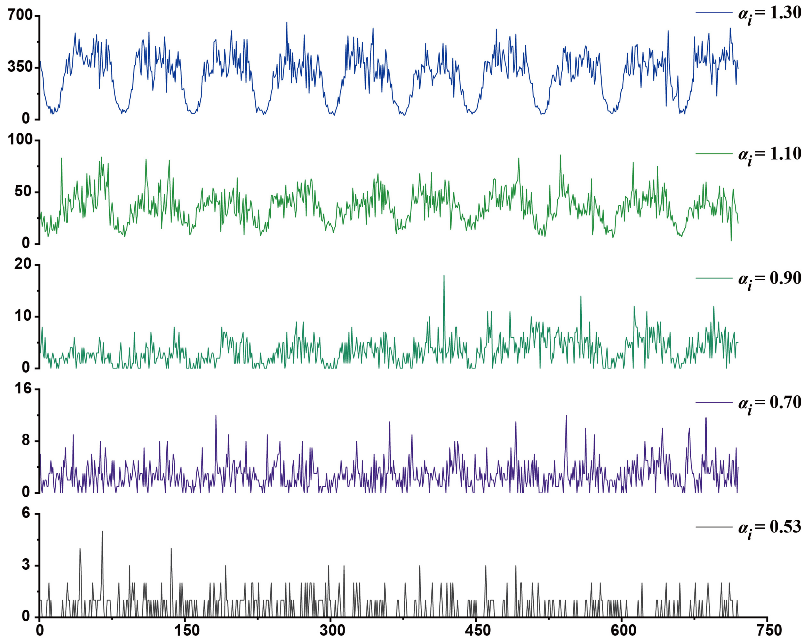

Fig. S12| Examples of time series of nominal population fluctuations (using mobile device data of grid cells in Beijing) characterized by different temporal scaling exponents α_i = 1.30, 1.10, 0.90, 0.70, 0.53. The horizontal axis represents the sequential number of time records arranged in order (totally 720 time records). The vertical axis represents the nominal population recorded by mobile device data. Generally, the higher the value of α_i, the larger the base of population movements (global rise-fall oscillations) and the smoother the fluctuations due to different strengths of long-term correlations (persistence).

Supplementary Note 5 (Identified locations of urban core functional areas or hot spots):

The patterns of the temporal scaling exponent α_i shed light on urban spatial organization and point to the cities' core functional areas or development hot spots. The identified locations of these areas are shown in Figs. S13–18. It is worth noting that as the core zones of urban economic development, the central business districts (CBDs) of five cities, Beijing CBD (Guomao), Shanghai Lujiazui CBD, Guangzhou Tianhe CBD (Pearl River new town), Shenzhen Futian CBD, and Boston CBD (Downtown), all present higher α_i values (marked in red). In addition, the main centres of urban development can also be accurately identified, such as the Forbidden City area, Wangfujing, Xidan and the Financial Street in Beijing's Dongcheng and Xicheng districts; People's Square in Shanghai; Pearl River new town in Guangzhou; Futian (Futian CBD) and Qianhai centre (Nanshan high-tech park) in Shenzhen, historical centre (Zone 1) in Milan, and Downtown Boston.

In Beijing, **the identified locations of urban core functional areas or hot spots** in Fig. S13 are listed as follows: 1 – Tian'anmen Square (the city centre of Beijing); 2 – Guomao (Beijing CBD); 3 – Zhongguancun science park (a national high-tech industrial development zone); 4 – Xidan (a famous commercial district and shopping centre); 5 – Financial Street (a gathering region of financial institution headquarters); 6 – Wangjing (The "second" CBD in Beijing); 7 – Yizhuang new town (a national economic and technological development zone); 8 – Embassy district; 9 – Shangdi (an information industry base); 10 – Beijing Olympic sports centre; 11 – Shougang high-end industry service area; 12 – Headquarters base; 13 – National Cultural Industry Innovation Zone; 14 – China International Exhibition Centre; and 15 – Airport logistics zone.

In Shanghai, **the identified locations of urban core functional areas or hot spots** in Fig. S14 are listed as follows: 1 – People's Square (the city centre of Shanghai); 2 – Lujiazui (Shanghai CBD); 3 – 'Sleepless City' and Shanghai railway station (a business subcentre and land transport hub); 4 – Zikawei (a famous commercial and shopping centre); 5 – Huamu–Longyang road city subcentre; 6 – Jinqiao city subcentre; 7 – Songjiang new town centre; 8 – Daning regional centre; 9 – Gubei regional centre; 10 – Zhenbei regional centre; 11 – Pujiang regional centre; 12 – Huinan regional centre; 13 – Jiading old town regional centre; 14 – Zhangjiang high-tech park; 15 – Caohejing development zone; 16 – Caohejing–Songjiang new industrial park; 17 – Nanqiao innovation zone; 18 – Zizhu high-tech zone; 19 – Airport economic zone; and 20 – Hongqiao Station.

In Guangzhou, **the identified locations of urban core functional areas or hot spots** in Fig. S15 are listed as follows: 1 – Pearl River new town (Guangzhou CBD and city centre); 2 – Pazhou Exhibition Centre; 3 – The People's Park (the old city centre of Guangzhou); 4 – Guangzhou east railway station; 5 – Wushan higher education district; 6 – Tianhe national software industry base; 7 – Beijing Road (a famous commercial and shopping centre); 8 – Martyrs' Memorial Park; 9 – Guangzhou railway station; 10 – Tianhe Smart City; 11 – Guangzhou Science City; 12 – Zhucun Machinery Parts City; 13 – Baigetan business district; 14 – Textile City; 15 – Haizhu industrial park; 16 – Guangzhou Wanbao industrial base; and 17 – Jinshan industrial park.

In Shenzhen, **the identified locations of urban core functional areas or hot spots** in Fig. S16 are listed as follows: 1 – Futian CBD; 2 – Nanshan high-tech park; and 3 – Futian Port.

In Milan, **the identified locations of urban core functional areas or hot spots** in Fig. S17 are listed as follows: 1 – Urban Centre - Comune di Milano; 2 – Milano Cadorna (a commuter railway station and a hub of public transport in the city centre); and 3 – Piazza degli Affari (the traditional financial centre of Milan).

In Greater Boston, **the identified locations of urban core functional areas or hot spots** in Fig. S18 are listed as follows: 1 – The Metropolitan Core (including districts such as Downtown Boston, Chinatown, High Spine, Back Bay, and major squares in Cambridge); 2 – Downtown Providence; 3 – Warwick Mall; 4 – Commercial Corridor along Rhode Island Route 2; 5 – Downtown Area in Worcester; 6 – Burlington Mall Area; 7 – Woburn Village; 8 – Hartwell Avenue Corridor in Lexington; 9 – Waltham Downtown; 10 – Waltham Technology Corridor; 11 – Shoppers World and the Golden Triangle (Framingham and Natick); 12 – Northshore Mall Area (Peabody and Danvers); 13 – Tuscan Village in Rockingham; 14 – Downtown Manchester; and 15 – Commercial and Retail Centers along Woodbury Avenue in Portsmouth.

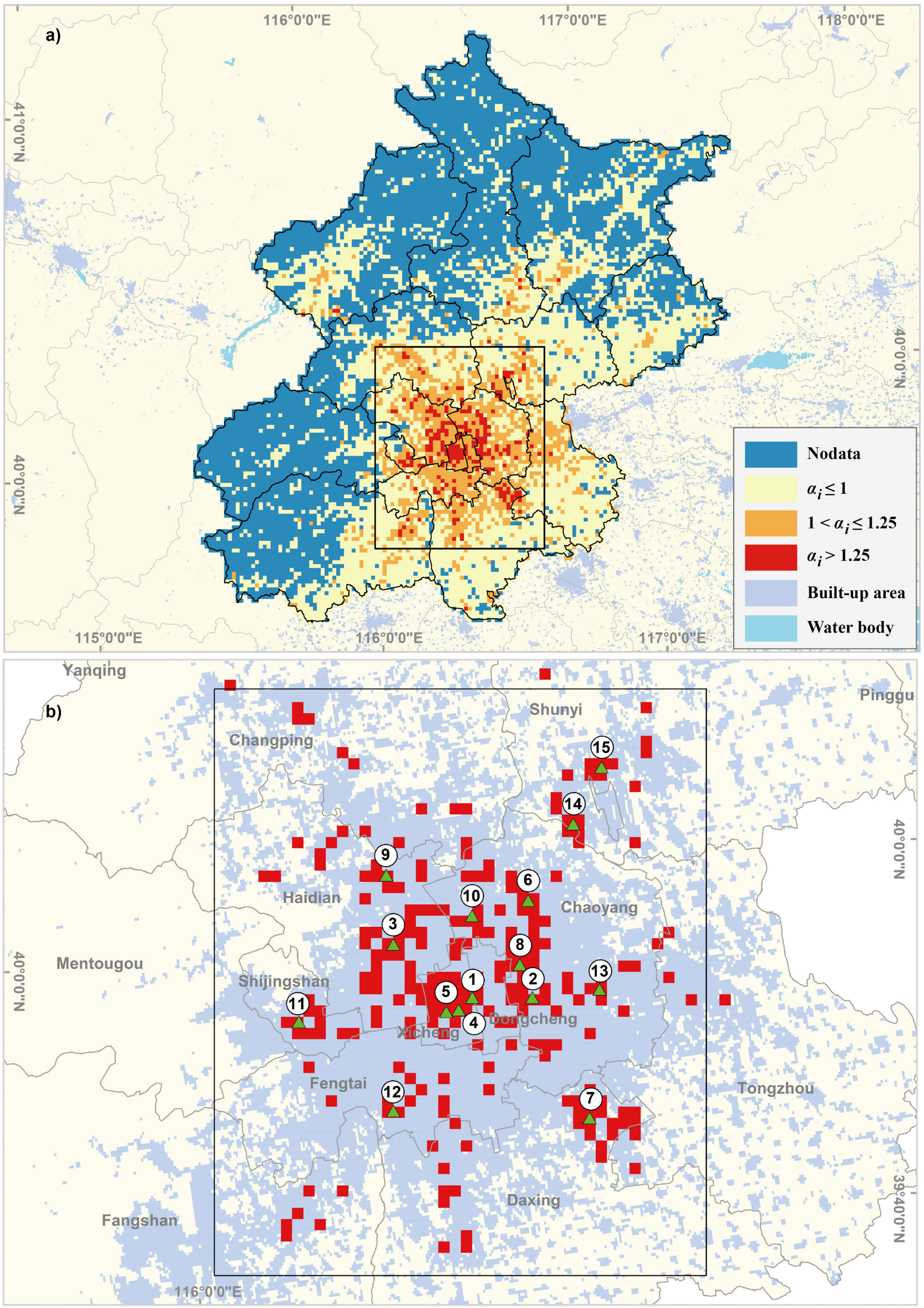

Fig. S13| Identification of urban hot spots in Beijing based on the value of temporal scaling exponents. a shows the urban structural features formed by three classified levels based on the value of temporal scaling exponents α_i. The black box in **a** contains the main clustering areas with high values of α_i. **b** zooms in the black box of **a** and exhibits several urban functional areas or hot spots (represented by small green triangles and labelled by numbers) and areas for which $\alpha_i > 1.25$ (in red). The names of these urban functional areas, or hot spots, are listed in Supplementary Note 5. [Note: This figure is Powered by Esri. The urban patches and water bodies on the base maps were extracted from the European Space Agency's 2018 Land Cover-Climate Change Initiative data[2] (© 2017 ESA Climate Change Initiative - Land Cover led by UCLouvain).]

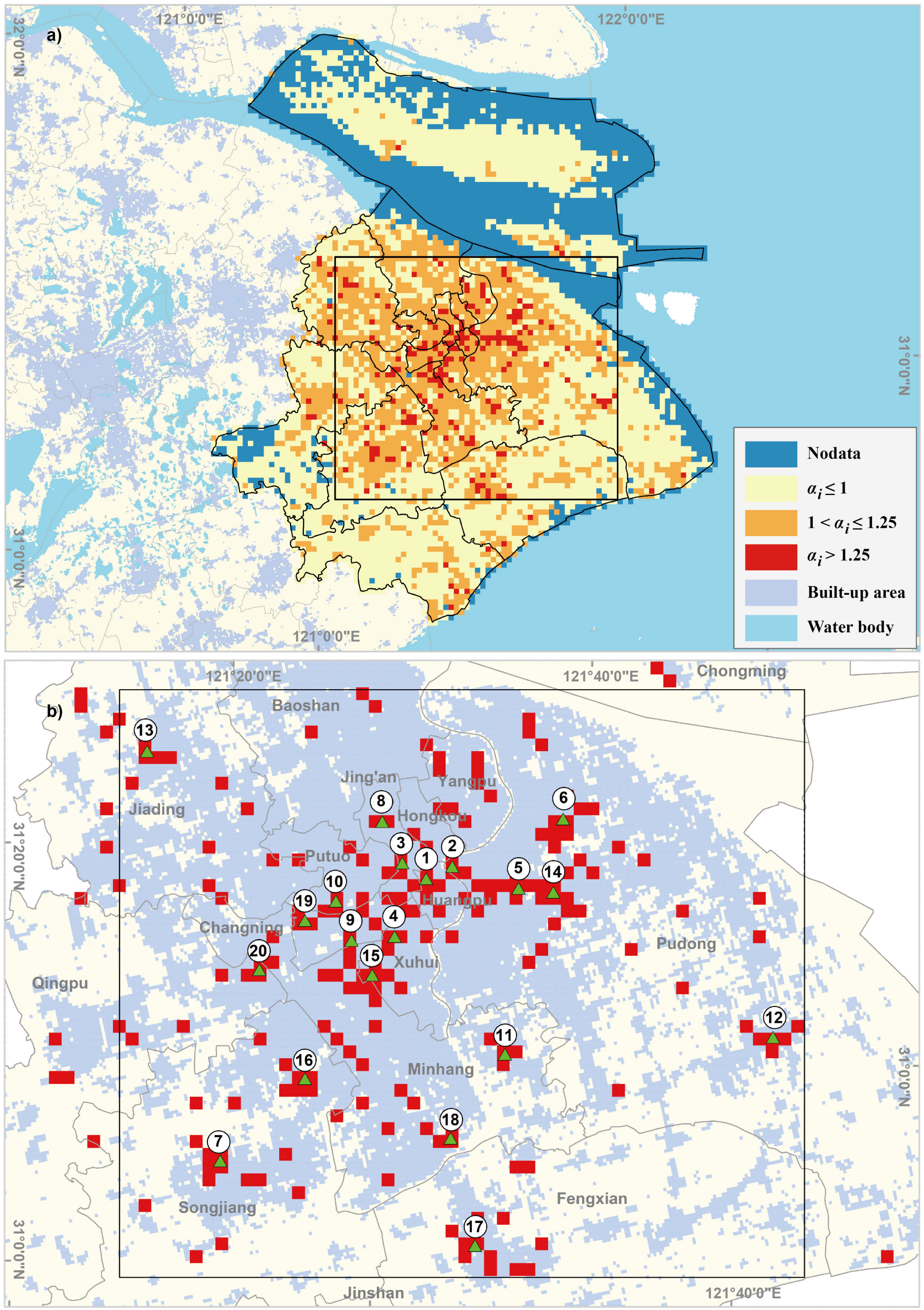

Fig. S14| Identification of urban hot spots in Shanghai based on the value of temporal scaling exponents. a shows the urban structural features formed by three classified levels based on the value of temporal scaling exponents α_i. The black box in **a** contains the main clustering areas with high values of α_i. **b** zooms in the black box of **a** and exhibits several urban functional areas or hot spots (represented by small green triangles and labelled by numbers) and areas for which $\alpha_i > 1.25$ (in red). The names of these urban functional areas, or hot spots, are listed in Supplementary Note 5. [Note: This figure is Powered by Esri. The urban patches and water bodies on the base maps were extracted from the European Space Agency's 2018 Land Cover-Climate Change Initiative data[2] (© 2017 ESA Climate Change Initiative - Land Cover led by UCLouvain).]

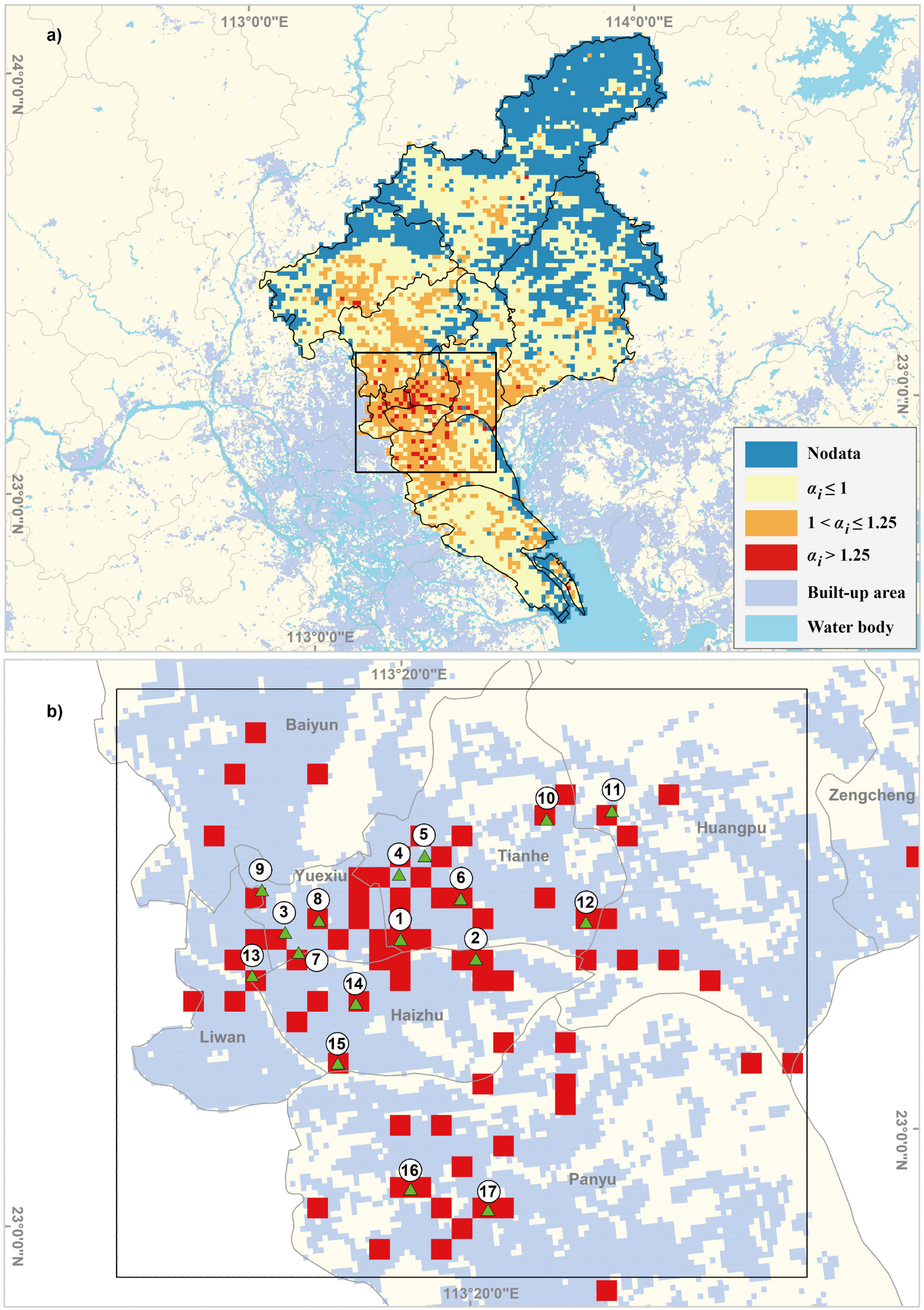

Fig. S15| Identification of urban hot spots in Guangzhou based on the value of temporal scaling exponents. a shows the urban structural features formed by three classified levels based on the value of temporal scaling exponents α_i. The black box in **a** contains the main clustering areas with high values of α_i. **b** zooms in the black box of **a** and exhibits several urban functional areas or hot spots (represented by small green triangles and labelled by numbers) and areas for which $\alpha_i > 1.25$ (in red). The names of these urban functional areas, or hot spots, are listed in Supplementary Note 5. [Note: This figure is Powered by Esri. The urban patches and water bodies on the base maps were extracted from the European Space Agency's 2018 Land Cover-Climate Change Initiative data[2] (© 2017 ESA Climate Change Initiative - Land Cover led by UCLouvain).]

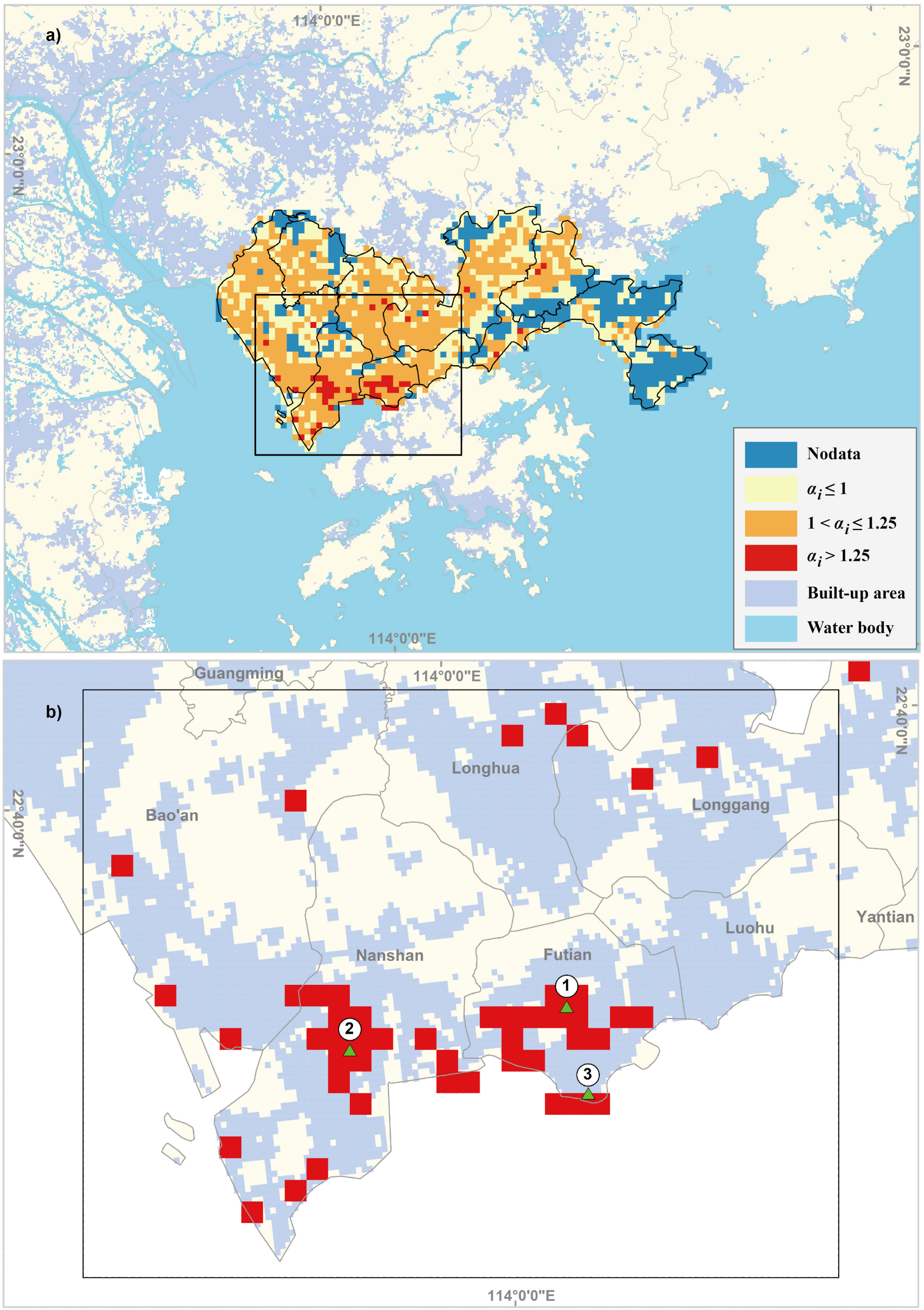

Fig. S16| Identification of urban hot spots in Shenzhen based on the value of temporal scaling exponents. a shows the urban structural features formed by three classified levels based on the value of temporal scaling exponents α_i. The black box in **a** contains the main clustering areas with high values of α_i. **b** zooms in the black box of **a** and exhibits several urban functional areas or hot spots (represented by small green triangles and labelled by numbers) and areas for which $\alpha_i > 1.25$ (in red). The names of these urban functional areas, or hot spots, are listed in Supplementary Note 5. [Note: This figure is Powered by Esri. The urban patches and water bodies on the base maps were extracted from the European Space Agency's 2018 Land Cover-Climate Change Initiative data[2] (© 2017 ESA Climate Change Initiative - Land Cover led by UCLouvain).]

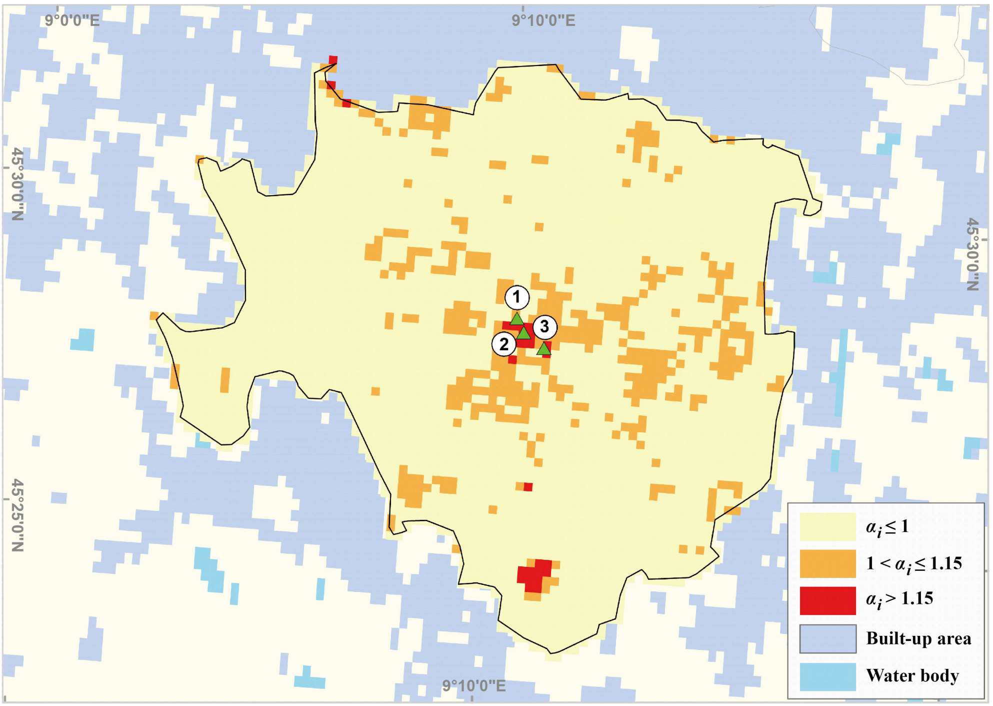

Fig. S17| Identification of urban hot spots in Milan based on the value of temporal scaling exponents. The figure shows the urban structural features formed by three classified levels based on the value of temporal scaling exponents and several urban functional areas or hot spots (represented by small green triangles and labelled by numbers) in the areas for which $\alpha_i > 1.15$. The names of these urban functional areas, or hot spots, are listed in Supplementary Note 5. [Note: This figure is Powered by Esri. The urban patches and water bodies on the base maps were extracted from the European Space Agency's 2013 Land Cover-Climate Change Initiative data[2] (© 2017 ESA Climate Change Initiative - Land Cover led by UCLouvain).]

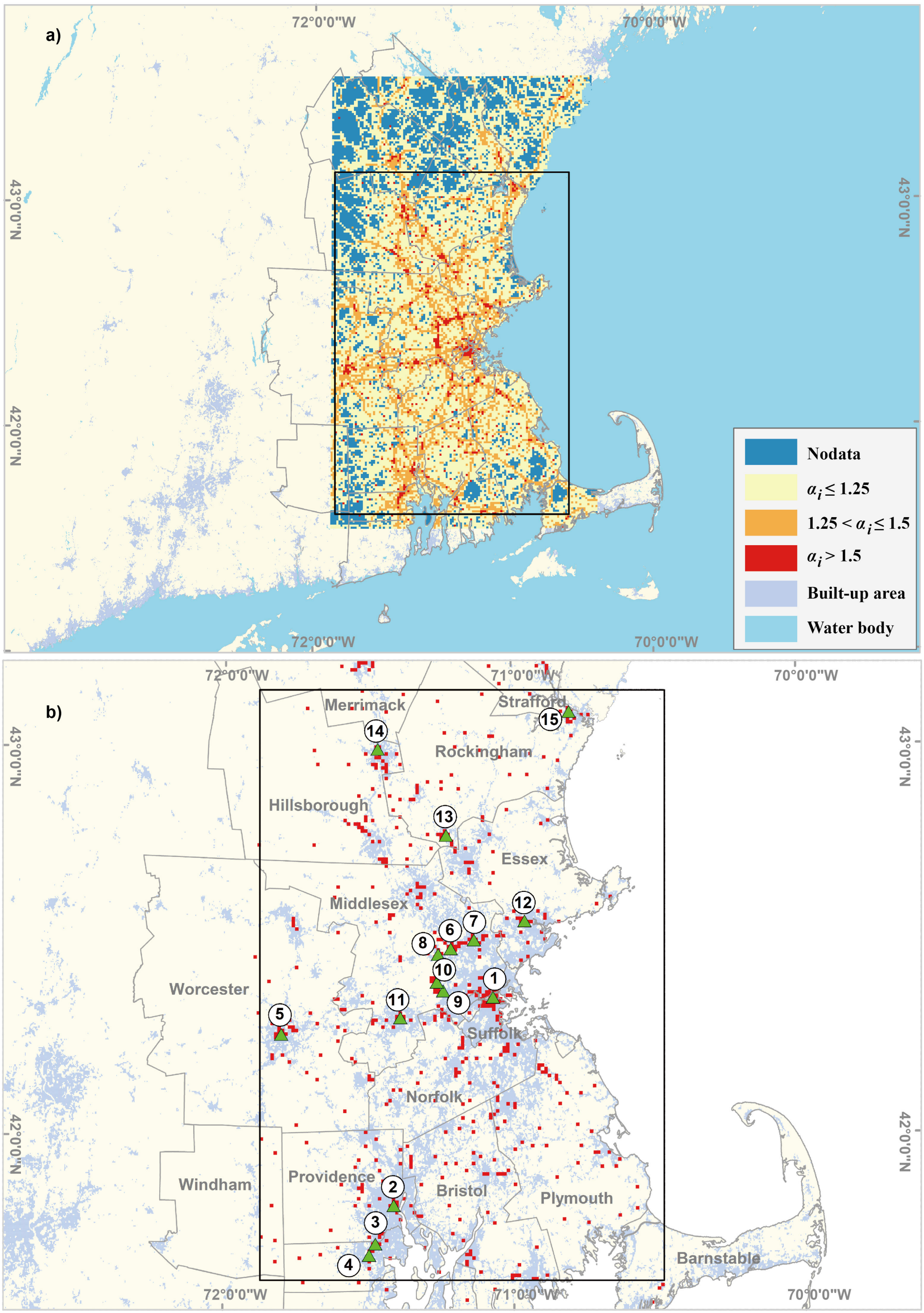

Fig. S18| Identification of urban hot spots in Greater Boston based on the value of temporal scaling exponents. **a** shows the urban structural features formed by three classified levels based on the value of temporal scaling exponents α_i. The black box in **a** contains the main clustering areas with high values of α_i. **b** zooms in the black box of **a** and exhibits several urban functional areas or hot spots (represented by small green triangles and labelled by numbers) and areas for which $\alpha_i > 1.5$ (in red). The names of these urban functional areas, or hot spots, are listed in Supplementary Note 5. [Note: This figure is Powered by Esri. The urban patches and water bodies on the base maps were extracted from the European Space Agency's 2020 Land Cover-Climate Change Initiative data[2] (© 2017 ESA Climate Change Initiative - Land Cover, led by UCLouvain).]

Supplementary Tables:

Table S3| The estimated temporal scaling exponent $\alpha(r)$ at a given distance r (km) from the closest city centre and the goodness of fit, R^2, for the six focal cases.

r	Beijing		Shanghai		Guangzhou		Shenzhen		Greater Boston		r	Milan	
	$\alpha(r)$	R^2	$\alpha(r)$	R^2	$\alpha(r)$	R^2	$\alpha(r)$	R^2	$\alpha(r)$	R^2		$\alpha(r)$	R^2
1.5	1.298	0.986	1.281	0.986	1.238	0.985	1.264	0.990	1.658	0.996	0.25	1.103	0.988
4.5	1.268	0.986	1.219	0.985	1.165	0.989	1.154	0.988	1.503	0.996	0.75	1.065	0.989
7.5	1.222	0.986	1.174	0.986	1.143	0.989	1.094	0.988	1.343	0.994	1.25	0.992	0.991
10.5	1.246	0.989	1.166	0.989	1.117	0.990	1.082	0.991	1.309	0.995	1.75	0.975	0.989
13.5	1.175	0.990	1.117	0.990	1.096	0.992	1.078	0.992	1.330	0.997	2.25	0.914	0.990
16.5	1.158	0.991	1.127	0.990	1.078	0.994	1.066	0.993	1.411	0.997	2.75	0.908	0.991
19.5	1.123	0.991	1.092	0.991	1.063	0.992	1.043	0.993	1.363	0.997	3.25	0.898	0.990
22.5	1.088	0.992	1.079	0.991	1.053	0.992	1.051	0.993	1.329	0.998	3.75	0.883	0.991
25.5	1.060	0.993	1.080	0.991	1.028	0.993	1.037	0.992	1.303	0.998	4.25	0.858	0.992
28.5	1.054	0.993	1.074	0.991	1.029	0.993	1.064	0.992	1.287	0.998	4.75	0.847	0.992
31.5	1.032	0.993	1.107	0.991	1.032	0.993	1.055	0.993	1.280	0.998	5.25	0.858	0.992
34.5	1.032	0.993	1.103	0.990	1.025	0.992	1.056	0.993	1.300	0.998	5.75	0.858	0.993
37.5	0.953	0.995	1.023	0.992	0.995	0.993	1.042	0.994	1.305	0.998	6.25	0.860	0.993
40.5	0.960	0.995	0.970	0.994	0.992	0.994	1.014	0.993	1.267	0.998	6.75	0.861	0.992
43.5	0.954	0.996	1.011	0.993	1.005	0.994	1.005	0.992	1.317	0.997	7.25	0.846	0.992
46.5	0.981	0.995	0.975	0.994	0.931	0.994	1.014	0.994	1.272	0.998	7.75	0.823	0.994
49.5	0.901	0.996	0.961	0.994	0.973	0.994	0.946	0.997	1.271	0.998	8.25	0.784	0.995
52.5	0.917	0.996	0.993	0.993	0.951	0.995	1.012	0.991	1.267	0.998	8.75	0.802	0.995
55.5	0.922	0.996	1.065	0.992	1.055	0.990	0.887	0.995	1.261	0.998	9.25	0.794	0.996
58.5	0.952	0.995	1.011	0.991	0.992	0.993	0.958	0.989	1.270	0.998	9.75	0.825	0.994
61.5	1.005	0.994	0.826	0.996	0.926	0.996	——	——	1.311	0.998	10.25	0.805	0.994
64.5	0.970	0.995	0.752	0.998	0.897	0.996	——	——	1.285	0.998	10.75	0.940	0.989
67.5	0.950	0.995	0.787	0.997	0.899	0.995	——	——	1.309	0.998	——	——	——
70.5	0.821	0.998	0.898	0.995	0.982	0.996	——	——	1.277	0.998	——	——	——
73.5	0.832	0.998	0.803	0.989	0.818	0.996	——	——	1.291	0.998	——	——	——
76.5	0.821	0.999	——	——	0.792	0.998	——	——	1.295	0.998	——	——	——
79.5	0.809	0.997	——	——	0.867	0.996	——	——	1.309	0.998	——	——	——
82.5	0.770	0.998	——	——	0.948	0.990	——	——	1.290	0.998	——	——	——
85.5	0.737	0.998	——	——	0.766	0.994	——	——	1.258	0.998	——	——	——
88.5	0.808	0.998	——	——	0.773	0.998	——	——	1.225	0.998	——	——	——
91.5	0.772	0.999	——	——	0.706	0.991	——	——	1.210	0.998	——	——	——
94.5	0.761	0.998	——	——	0.705	0.997	——	——	1.234	0.998	——	——	——
97.5	0.749	0.998	——	——	0.709	0.997	——	——	1.214	0.998	——	——	——
100.5	0.746	0.997	——	——	0.744	0.998	——	——	1.290	0.997	——	——	——
103.5	0.767	0.999	——	——	0.903	0.996	——	——	1.256	0.997	——	——	——
106.5	0.964	0.993	——	——	0.870	0.998	——	——	1.203	0.998	——	——	——
109.5	0.823	0.995	——	——	0.682	0.998	——	——	1.152	0.997	——	——	——
112.5	0.777	0.998	——	——	0.726	0.996	——	——	1.124	0.997	——	——	——
115.5	0.779	0.973	——	——	0.730	0.993	——	——	1.100	0.997	——	——	——
118.5	0.737	0.998	——	——	0.706	0.990	——	——	1.123	0.996	——	——	——
121.5	0.615	0.993	——	——	——	——	——	——	1.165	0.997	——	——	——
124.5	0.611	0.992	——	——	——	——	——	——	1.130	0.997	——	——	——
127.5	——	——	——	——	——	——	——	——	1.200	0.996	——	——	——
130.5	——	——	——	——	——	——	——	——	1.178	0.996	——	——	——
133.5	——	——	——	——	——	——	——	——	1.217	0.997	——	——	——
136.5	——	——	——	——	——	——	——	——	1.219	0.997	——	——	——
139.5	——	——	——	——	——	——	——	——	1.214	0.998	——	——	——

142.5	——	——	——	——	——	——	——	——	1.203	0.998	——	——	——
145.5	——	——	——	——	——	——	——	——	1.167	0.998	——	——	——

[Note: The distance interval of each case is determined considering its size (to ensure sufficient distance samples) and spatial resolution of the original data (to ensure the representativeness and accuracy of samples).]

Table S4| The estimated spatial scaling exponent of population fluctuations $d(s)$ at a given temporal scale s and the goodness of fit, R^2, for the six focal cases.

s	Beijing		Shanghai		Guangzhou		Shenzhen ($r \leq 25.5$km)		Shenzhen ($r > 25.5$km)		Milan		Greater Boston	
	$d(s)$	R^2	$d(s)$	R^2	$d(s)$	R^2	$d(s)$	R^2	$d(s)$	R^2	$d(s)$	R^2	$d(s)$	R^2
3	-1.713	0.960	-1.833	0.923	-1.811	0.945	-0.262	0.857	-5.556	0.898	-1.288	0.944	-0.537	0.944
4	-1.717	0.963	-1.830	0.923	-1.803	0.945	-0.267	0.859	-5.508	0.897	-1.285	0.944	-0.565	0.941
5	-1.714	0.956	-1.830	0.921	-1.815	0.941	-0.259	0.854	-5.555	0.898	-1.286	0.946	-0.595	0.944
6	-1.712	0.955	-1.835	0.921	-1.810	0.945	-0.261	0.848	-5.545	0.900	-1.291	0.947	-0.617	0.944
7	-1.716	0.955	-1.835	0.920	-1.812	0.941	-0.261	0.855	-5.530	0.900	-1.283	0.946	-0.646	0.942
8	-1.726	0.954	-1.840	0.918	-1.819	0.941	-0.249	0.838	-5.566	0.898	-1.293	0.947	-0.666	0.935
9	-1.728	0.952	-1.845	0.919	-1.823	0.942	-0.262	0.844	-5.598	0.901	-1.294	0.947	-0.683	0.942
10	-1.747	0.952	-1.857	0.916	-1.835	0.940	-0.266	0.846	-5.600	0.900	-1.290	0.948	-0.701	0.939
11	-1.755	0.952	-1.865	0.916	-1.839	0.939	-0.268	0.846	-5.604	0.900	-1.295	0.949	-0.720	0.937
12	-1.763	0.950	-1.874	0.914	-1.850	0.937	-0.250	0.834	-5.622	0.897	-1.297	0.948	-0.741	0.929
13	-1.785	0.949	-1.885	0.915	-1.861	0.938	-0.276	0.847	-5.629	0.900	-1.305	0.950	-0.744	0.936
14	-1.796	0.950	-1.897	0.914	-1.877	0.937	-0.279	0.847	-5.661	0.900	-1.307	0.952	-0.753	0.935
15	-1.812	0.948	-1.914	0.912	-1.894	0.935	-0.284	0.848	-5.717	0.898	-1.306	0.950	-0.763	0.934
16	-1.830	0.948	-1.919	0.911	-1.900	0.936	-0.285	0.850	-5.728	0.899	-1.337	0.954	-0.769	0.933
17	-1.839	0.949	-1.934	0.911	-1.906	0.935	-0.294	0.852	-5.720	0.899	-1.314	0.953	-0.774	0.933
18	-1.808	0.948	-1.917	0.920	-1.896	0.938	-0.354	0.887	-5.607	0.906	-1.280	0.948	-0.753	0.930
19	-1.880	0.948	-1.962	0.908	-1.933	0.933	-0.303	0.854	-5.763	0.899	-1.321	0.954	-0.793	0.931
20	-1.887	0.948	-1.975	0.908	-1.948	0.933	-0.301	0.854	-5.833	0.896	-1.341	0.955	-0.787	0.931
21	-1.896	0.948	-1.981	0.906	-1.958	0.932	-0.308	0.858	-5.804	0.897	-1.333	0.955	-0.797	0.930
22	-1.910	0.948	-1.998	0.905	-1.976	0.932	-0.315	0.863	-5.845	0.898	-1.345	0.958	-0.797	0.931
23	-1.914	0.948	-1.995	0.906	-1.971	0.932	-0.323	0.864	-5.817	0.898	-1.354	0.958	-0.804	0.929
24	-1.811	0.946	-1.916	0.911	-1.901	0.935	-0.343	0.877	-5.582	0.899	-1.295	0.943	-0.803	0.926
25	-1.936	0.946	-2.017	0.903	-1.991	0.930	-0.332	0.872	-5.835	0.899	-1.370	0.959	-0.812	0.928
26	-1.955	0.946	-2.031	0.903	-2.008	0.930	-0.333	0.870	-5.877	0.896	-1.365	0.957	-0.809	0.930
27	-1.964	0.946	-2.037	0.902	-2.015	0.930	-0.337	0.873	-5.868	0.896	-1.367	0.957	-0.817	0.928
28	-1.978	0.948	-2.048	0.901	-2.028	0.929	-0.340	0.876	-5.908	0.897	-1.368	0.958	-0.819	0.928
29	-1.997	0.948	-2.057	0.900	-2.036	0.927	-0.343	0.878	-5.943	0.895	-1.401	0.960	-0.826	0.927
30	-2.002	0.946	-2.077	0.898	-2.058	0.929	-0.345	0.875	-5.964	0.895	-1.403	0.961	-0.814	0.928
31	-2.007	0.947	-2.077	0.898	-2.054	0.928	-0.350	0.882	-5.942	0.896	-1.383	0.959	-0.823	0.927
32	-2.018	0.946	-2.080	0.899	-2.064	0.928	-0.356	0.883	-5.949	0.896	-1.397	0.959	-0.823	0.927
33	-2.027	0.945	-2.092	0.896	-2.082	0.926	-0.354	0.885	-5.996	0.894	-1.376	0.959	-0.825	0.925
34	-2.036	0.946	-2.094	0.897	-2.070	0.928	-0.363	0.887	-5.945	0.897	-1.427	0.961	-0.825	0.926
35	-2.042	0.945	-2.101	0.896	-2.084	0.926	-0.362	0.888	-5.984	0.896	-1.412	0.960	-0.826	0.926
36	-2.041	0.948	-2.077	0.899	-2.053	0.933	-0.393	0.889	-5.855	0.898	-1.429	0.956	-0.831	0.924
37	-2.056	0.946	-2.112	0.895	-2.099	0.926	-0.369	0.891	-5.996	0.896	-1.421	0.960	-0.828	0.925
38	-2.070	0.947	-2.118	0.894	-2.094	0.926	-0.370	0.891	-6.014	0.897	-1.445	0.961	-0.831	0.924
39	-2.069	0.946	-2.121	0.895	-2.115	0.925	-0.376	0.892	-6.007	0.897	-1.427	0.959	-0.831	0.925
40	-2.090	0.946	-2.129	0.894	-2.120	0.926	-0.371	0.892	-6.025	0.895	-1.435	0.957	-0.830	0.924
41	-2.086	0.946	-2.128	0.894	-2.122	0.925	-0.381	0.895	-6.005	0.897	-1.435	0.960	-0.832	0.923
42	-2.093	0.946	-2.138	0.893	-2.126	0.923	-0.382	0.897	-6.032	0.897	-1.427	0.960	-0.831	0.925
43	-2.098	0.947	-2.138	0.893	-2.122	0.924	-0.389	0.898	-6.008	0.898	-1.434	0.960	-0.834	0.924
44	-2.099	0.945	-2.140	0.893	-2.134	0.923	-0.389	0.899	-6.026	0.898	-1.448	0.959	-0.832	0.923
45	-2.113	0.945	-2.149	0.891	-2.142	0.923	-0.386	0.899	-6.049	0.897	-1.460	0.958	-0.830	0.924
46	-2.114	0.945	-2.153	0.891	-2.143	0.923	-0.393	0.899	-6.039	0.898	-1.452	0.959	-0.834	0.923
47	-2.109	0.944	-2.144	0.892	-2.140	0.923	-0.398	0.904	-6.012	0.900	-1.452	0.959	-0.835	0.922
48	-2.131	0.944	-2.164	0.890	-2.161	0.921	-0.391	0.905	-6.075	0.896	-1.489	0.961	-0.837	0.922
49	-2.122	0.945	-2.153	0.891	-2.150	0.923	-0.401	0.905	-6.034	0.899	-1.469	0.958	-0.834	0.921
50	-2.128	0.945	-2.158	0.891	-2.155	0.923	-0.405	0.905	-6.014	0.900	-1.459	0.959	-0.837	0.921
51	-2.132	0.945	-2.159	0.890	-2.157	0.922	-0.405	0.906	-6.015	0.901	-1.451	0.960	-0.842	0.921

52	-2.137	0.944	-2.164	0.889	-2.163	0.922	-0.403	0.905	-6.030	0.900	-1.469	0.959	-0.845	0.920
53	-2.136	0.946	-2.163	0.890	-2.160	0.922	-0.412	0.906	-6.018	0.902	-1.470	0.958	-0.837	0.920
54	-2.141	0.945	-2.163	0.891	-2.153	0.922	-0.417	0.912	-5.994	0.901	-1.464	0.959	-0.834	0.920
55	-2.153	0.944	-2.168	0.891	-2.166	0.922	-0.417	0.913	-6.041	0.902	-1.443	0.957	-0.837	0.919
56	-2.153	0.943	-2.171	0.889	-2.172	0.921	-0.414	0.912	-6.047	0.902	-1.459	0.957	-0.843	0.918
57	-2.157	0.944	-2.171	0.890	-2.167	0.920	-0.419	0.912	-6.027	0.902	-1.468	0.957	-0.840	0.918
58	-2.157	0.947	-2.171	0.891	-2.164	0.922	-0.426	0.912	-5.994	0.903	-1.471	0.958	-0.834	0.919
59	-2.157	0.947	-2.176	0.891	-2.170	0.922	-0.426	0.912	-5.995	0.904	-1.469	0.960	-0.830	0.920
60	-2.163	0.947	-2.179	0.890	-2.175	0.922	-0.421	0.913	-6.011	0.903	-1.459	0.960	-0.832	0.920
61	-2.166	0.945	-2.182	0.888	-2.180	0.921	-0.420	0.912	-6.029	0.902	-1.463	0.959	-0.837	0.919
62	-2.167	0.944	-2.182	0.889	-2.184	0.920	-0.427	0.914	-6.008	0.904	-1.470	0.958	-0.836	0.918
63	-2.166	0.944	-2.177	0.890	-2.171	0.921	-0.437	0.916	-5.970	0.907	-1.483	0.957	-0.835	0.917
64	-2.169	0.944	-2.177	0.891	-2.173	0.921	-0.444	0.917	-5.959	0.908	-1.490	0.957	-0.833	0.917
65	-2.179	0.944	-2.185	0.890	-2.188	0.920	-0.438	0.917	-5.994	0.907	-1.507	0.958	-0.833	0.917
66	-2.180	0.943	-2.186	0.889	-2.189	0.920	-0.433	0.915	-6.000	0.906	-1.502	0.958	-0.839	0.916
67	-2.187	0.944	-2.190	0.888	-2.191	0.920	-0.430	0.916	-6.017	0.906	-1.497	0.958	-0.839	0.917
68	-2.182	0.944	-2.187	0.889	-2.188	0.920	-0.435	0.917	-6.004	0.907	-1.485	0.957	-0.837	0.917
69	-2.176	0.944	-2.182	0.890	-2.180	0.921	-0.446	0.918	-5.970	0.908	-1.480	0.956	-0.834	0.916
70	-2.166	0.945	-2.172	0.893	-2.167	0.921	-0.464	0.920	-5.904	0.912	-1.479	0.955	-0.828	0.916
71	-2.176	0.945	-2.185	0.893	-2.177	0.919	-0.469	0.922	-5.909	0.914	-1.503	0.957	-0.824	0.915
72	-2.191	0.946	-2.199	0.891	-2.191	0.918	-0.462	0.922	-5.933	0.913	-1.534	0.960	-0.825	0.915

[Note: The column of temporal scale s lists the number of temporal units. One temporal unit represents 20 minutes. For example, the value of 3 represents a temporal scale of $3 \times 20 = 60$ minutes.]

Table S5| The estimated allometric exponent $A(s)$ of population fluctuations and population density at a given temporal scale s and the goodness of fit, R^2, for the six focal cases.

s	Beijing		Shanghai		Guangzhou		Shenzhen		Milan		Greater Boston	
	$A(s)$	R^2	$A(s)$	R^2	$A(s)$	R^2	$A(s)$	R^2	$A(s)$	R^2	$A(s)$	R^2
3	0.636	0.895	0.877	0.958	0.777	0.913	0.744	0.949	0.413	0.796	0.671	0.826
4	0.637	0.897	0.876	0.957	0.778	0.914	0.739	0.950	0.413	0.802	0.707	0.824
5	0.636	0.895	0.876	0.957	0.778	0.915	0.742	0.950	0.412	0.798	0.746	0.830
6	0.635	0.892	0.878	0.957	0.776	0.913	0.741	0.950	0.413	0.797	0.775	0.834
7	0.636	0.893	0.877	0.957	0.777	0.914	0.739	0.950	0.411	0.800	0.814	0.835
8	0.640	0.894	0.879	0.956	0.778	0.914	0.739	0.947	0.413	0.799	0.841	0.834
9	0.641	0.892	0.881	0.955	0.785	0.915	0.746	0.949	0.414	0.802	0.858	0.832
10	0.647	0.893	0.887	0.954	0.784	0.914	0.746	0.951	0.412	0.796	0.886	0.838
11	0.650	0.892	0.890	0.955	0.787	0.915	0.746	0.949	0.412	0.797	0.909	0.836
12	0.652	0.892	0.891	0.955	0.787	0.914	0.744	0.945	0.413	0.794	0.933	0.824
13	0.660	0.891	0.898	0.954	0.795	0.915	0.749	0.950	0.414	0.796	0.940	0.836
14	0.665	0.892	0.903	0.953	0.801	0.915	0.753	0.950	0.413	0.793	0.952	0.836
15	0.671	0.891	0.909	0.953	0.806	0.915	0.760	0.950	0.414	0.794	0.963	0.833
16	0.676	0.891	0.913	0.953	0.810	0.916	0.761	0.950	0.421	0.794	0.971	0.833
17	0.680	0.892	0.920	0.952	0.814	0.915	0.762	0.950	0.415	0.793	0.978	0.834
18	0.673	0.886	0.926	0.953	0.821	0.914	0.767	0.959	0.409	0.803	0.945	0.818
19	0.694	0.892	0.934	0.952	0.825	0.916	0.768	0.951	0.415	0.787	1.002	0.832
20	0.698	0.892	0.936	0.952	0.828	0.915	0.776	0.950	0.421	0.792	0.996	0.833
21	0.700	0.891	0.940	0.951	0.831	0.915	0.774	0.950	0.418	0.791	1.007	0.832
22	0.707	0.891	0.949	0.950	0.838	0.915	0.780	0.952	0.420	0.790	1.009	0.835
23	0.706	0.892	0.946	0.951	0.835	0.915	0.778	0.952	0.422	0.787	1.016	0.831
24	0.670	0.891	0.911	0.954	0.809	0.914	0.755	0.955	0.419	0.814	1.008	0.816
25	0.715	0.891	0.957	0.950	0.845	0.916	0.782	0.953	0.426	0.788	1.028	0.830
26	0.722	0.891	0.962	0.950	0.848	0.915	0.787	0.952	0.426	0.787	1.023	0.833
27	0.725	0.890	0.966	0.949	0.851	0.916	0.787	0.952	0.427	0.792	1.033	0.831
28	0.730	0.892	0.971	0.949	0.855	0.915	0.792	0.953	0.427	0.788	1.036	0.831
29	0.737	0.894	0.975	0.948	0.859	0.915	0.796	0.952	0.434	0.789	1.045	0.829
30	0.738	0.890	0.982	0.948	0.865	0.915	0.799	0.952	0.437	0.790	1.032	0.834
31	0.741	0.891	0.984	0.947	0.865	0.915	0.798	0.954	0.431	0.790	1.044	0.835
32	0.744	0.891	0.986	0.948	0.869	0.916	0.800	0.954	0.436	0.794	1.043	0.832
33	0.747	0.890	0.991	0.947	0.871	0.915	0.804	0.953	0.429	0.793	1.045	0.830
34	0.751	0.891	0.992	0.946	0.875	0.916	0.802	0.955	0.442	0.790	1.048	0.835
35	0.753	0.891	0.995	0.947	0.874	0.915	0.805	0.954	0.438	0.792	1.048	0.833
36	0.756	0.892	0.991	0.946	0.872	0.920	0.798	0.959	0.449	0.797	1.057	0.835
37	0.758	0.890	1.001	0.947	0.880	0.915	0.808	0.954	0.442	0.792	1.051	0.833
38	0.763	0.892	1.004	0.946	0.884	0.916	0.811	0.955	0.448	0.791	1.056	0.835
39	0.763	0.891	1.005	0.946	0.884	0.915	0.811	0.955	0.444	0.795	1.055	0.834
40	0.770	0.892	1.007	0.945	0.887	0.915	0.813	0.955	0.449	0.796	1.055	0.835
41	0.769	0.891	1.011	0.945	0.887	0.915	0.812	0.956	0.447	0.795	1.058	0.834
42	0.772	0.890	1.014	0.945	0.889	0.915	0.816	0.956	0.444	0.795	1.057	0.836
43	0.774	0.892	1.014	0.945	0.890	0.916	0.814	0.957	0.446	0.796	1.060	0.835
44	0.774	0.890	1.017	0.945	0.892	0.915	0.817	0.957	0.452	0.797	1.058	0.835
45	0.778	0.890	1.020	0.945	0.893	0.914	0.819	0.957	0.456	0.795	1.057	0.837
46	0.779	0.891	1.023	0.945	0.895	0.915	0.819	0.957	0.454	0.798	1.062	0.836
47	0.778	0.888	1.021	0.945	0.895	0.914	0.818	0.958	0.454	0.799	1.062	0.834
48	0.785	0.888	1.027	0.943	0.898	0.914	0.823	0.956	0.464	0.798	1.067	0.837
49	0.782	0.889	1.024	0.944	0.898	0.915	0.821	0.958	0.460	0.801	1.062	0.836
50	0.784	0.890	1.028	0.944	0.900	0.916	0.819	0.959	0.455	0.799	1.065	0.835
51	0.786	0.889	1.029	0.943	0.901	0.914	0.820	0.960	0.452	0.798	1.070	0.833
52	0.788	0.889	1.030	0.943	0.902	0.914	0.822	0.959	0.459	0.801	1.074	0.833
53	0.788	0.889	1.031	0.944	0.902	0.915	0.822	0.960	0.461	0.803	1.067	0.835

54	0.789	0.890	1.030	0.944	0.900	0.916	0.819	0.960	0.457	0.799	1.063	0.837
55	0.792	0.888	1.035	0.944	0.905	0.915	0.825	0.960	0.452	0.799	1.067	0.835
56	0.792	0.888	1.036	0.944	0.905	0.915	0.825	0.960	0.457	0.802	1.074	0.833
57	0.795	0.889	1.036	0.944	0.905	0.914	0.824	0.961	0.461	0.805	1.070	0.835
58	0.796	0.890	1.036	0.944	0.906	0.916	0.822	0.961	0.461	0.804	1.064	0.837
59	0.796	0.890	1.037	0.944	0.907	0.915	0.823	0.961	0.457	0.796	1.060	0.838
60	0.797	0.889	1.037	0.943	0.909	0.915	0.823	0.960	0.454	0.793	1.062	0.838
61	0.798	0.889	1.040	0.943	0.909	0.914	0.825	0.960	0.457	0.797	1.067	0.837
62	0.798	0.889	1.041	0.943	0.909	0.914	0.825	0.961	0.460	0.800	1.067	0.836
63	0.798	0.888	1.042	0.943	0.909	0.915	0.823	0.963	0.466	0.806	1.067	0.836
64	0.799	0.888	1.042	0.944	0.911	0.916	0.823	0.963	0.469	0.809	1.064	0.837
65	0.802	0.888	1.044	0.943	0.912	0.915	0.826	0.963	0.472	0.803	1.065	0.837
66	0.802	0.888	1.043	0.943	0.912	0.915	0.825	0.962	0.471	0.803	1.071	0.835
67	0.805	0.889	1.045	0.943	0.914	0.915	0.827	0.962	0.469	0.803	1.071	0.835
68	0.804	0.889	1.046	0.943	0.913	0.915	0.827	0.963	0.467	0.805	1.069	0.836
69	0.802	0.887	1.047	0.943	0.911	0.915	0.825	0.964	0.467	0.809	1.067	0.838
70	0.800	0.887	1.046	0.943	0.910	0.915	0.822	0.965	0.468	0.814	1.060	0.839
71	0.804	0.886	1.051	0.943	0.915	0.915	0.825	0.966	0.474	0.812	1.056	0.841
72	0.808	0.887	1.055	0.943	0.917	0.915	0.826	0.965	0.480	0.806	1.058	0.842

[Note: The column of temporal scale s lists the number of temporal units. One temporal unit represents 20 minutes. For example, the value of 3 represents a temporal scale of $3 \times 20 = 60$ minutes.]

Table S6| The estimated allometric exponent $A(s)$ of population fluctuations and the density of POI at a given temporal scale s and the goodness of fit, R^2, for the six focal cases.

s	Beijing		Shanghai		Guangzhou		Shenzhen		Milan		Greater Boston	
	$A(s)$	R^2	$A(s)$	R^2	$A(s)$	R^2	$A(s)$	R^2	$A(s)$	R^2	$A(s)$	R^2
3	0.789	0.892	0.816	0.897	0.611	0.889	0.898	0.913	0.724	0.912	0.332	0.929
4	0.788	0.893	0.815	0.896	0.609	0.889	0.892	0.914	0.717	0.912	0.350	0.929
5	0.790	0.893	0.815	0.896	0.613	0.887	0.895	0.913	0.719	0.912	0.368	0.929
6	0.791	0.891	0.817	0.896	0.611	0.889	0.894	0.913	0.727	0.917	0.382	0.932
7	0.791	0.892	0.816	0.896	0.611	0.886	0.891	0.912	0.728	0.919	0.401	0.931
8	0.795	0.892	0.818	0.894	0.613	0.888	0.890	0.908	0.734	0.920	0.415	0.933
9	0.797	0.891	0.821	0.894	0.616	0.889	0.899	0.910	0.733	0.919	0.423	0.930
10	0.804	0.890	0.825	0.893	0.619	0.888	0.899	0.912	0.736	0.924	0.435	0.929
11	0.807	0.890	0.828	0.892	0.621	0.889	0.899	0.911	0.746	0.928	0.447	0.931
12	0.808	0.887	0.827	0.890	0.623	0.889	0.895	0.905	0.744	0.925	0.462	0.931
13	0.819	0.888	0.835	0.891	0.628	0.889	0.903	0.911	0.759	0.932	0.462	0.930
14	0.825	0.888	0.840	0.891	0.633	0.889	0.908	0.910	0.763	0.933	0.468	0.930
15	0.831	0.886	0.845	0.890	0.639	0.888	0.916	0.911	0.765	0.935	0.474	0.928
16	0.837	0.885	0.849	0.889	0.641	0.890	0.917	0.910	0.791	0.941	0.479	0.931
17	0.842	0.885	0.855	0.889	0.644	0.889	0.918	0.912	0.778	0.939	0.481	0.929
18	0.840	0.892	0.867	0.899	0.645	0.889	0.929	0.928	0.724	0.920	0.468	0.921
19	0.857	0.883	0.867	0.887	0.653	0.888	0.926	0.912	0.798	0.944	0.494	0.930
20	0.863	0.883	0.869	0.887	0.658	0.891	0.935	0.911	0.803	0.945	0.490	0.929
21	0.865	0.882	0.873	0.885	0.660	0.889	0.932	0.912	0.801	0.944	0.497	0.930
22	0.873	0.882	0.881	0.885	0.667	0.889	0.941	0.914	0.815	0.948	0.497	0.930
23	0.872	0.881	0.878	0.884	0.665	0.889	0.938	0.915	0.824	0.950	0.501	0.930
24	0.829	0.882	0.845	0.889	0.643	0.890	0.913	0.921	0.748	0.928	0.502	0.930
25	0.882	0.880	0.887	0.883	0.672	0.887	0.943	0.916	0.839	0.951	0.507	0.930
26	0.889	0.879	0.893	0.883	0.678	0.890	0.949	0.915	0.841	0.951	0.504	0.930
27	0.894	0.878	0.897	0.883	0.680	0.889	0.949	0.916	0.838	0.950	0.510	0.930
28	0.899	0.878	0.901	0.883	0.685	0.891	0.955	0.915	0.839	0.950	0.511	0.930
29	0.904	0.877	0.905	0.882	0.688	0.889	0.959	0.914	0.863	0.954	0.516	0.929
30	0.907	0.876	0.909	0.881	0.695	0.893	0.964	0.915	0.862	0.952	0.508	0.931
31	0.913	0.877	0.913	0.880	0.693	0.889	0.963	0.917	0.858	0.954	0.514	0.930
32	0.916	0.876	0.915	0.882	0.697	0.891	0.965	0.917	0.862	0.953	0.514	0.931
33	0.918	0.874	0.919	0.880	0.703	0.891	0.970	0.916	0.855	0.954	0.516	0.930
34	0.924	0.877	0.922	0.880	0.701	0.892	0.968	0.919	0.887	0.957	0.516	0.931
35	0.925	0.875	0.923	0.880	0.704	0.890	0.972	0.918	0.878	0.956	0.517	0.931
36	0.931	0.880	0.923	0.883	0.697	0.891	0.963	0.924	0.882	0.951	0.520	0.931
37	0.931	0.875	0.928	0.880	0.709	0.891	0.975	0.918	0.883	0.955	0.518	0.931
38	0.936	0.875	0.932	0.880	0.709	0.892	0.978	0.918	0.900	0.956	0.520	0.931
39	0.937	0.874	0.933	0.880	0.715	0.892	0.979	0.920	0.887	0.955	0.520	0.931
40	0.943	0.873	0.936	0.878	0.717	0.893	0.981	0.918	0.893	0.954	0.520	0.931
41	0.944	0.874	0.938	0.879	0.718	0.892	0.981	0.920	0.892	0.955	0.521	0.931
42	0.946	0.873	0.940	0.878	0.720	0.892	0.985	0.921	0.887	0.955	0.520	0.932
43	0.948	0.873	0.941	0.879	0.719	0.892	0.983	0.921	0.892	0.955	0.522	0.932
44	0.949	0.873	0.945	0.880	0.723	0.893	0.987	0.922	0.901	0.955	0.521	0.931
45	0.952	0.873	0.947	0.879	0.726	0.896	0.989	0.922	0.909	0.954	0.520	0.932
46	0.955	0.873	0.950	0.879	0.727	0.894	0.989	0.922	0.905	0.955	0.522	0.932
47	0.955	0.873	0.949	0.879	0.725	0.892	0.987	0.923	0.902	0.955	0.523	0.931
48	0.962	0.871	0.953	0.877	0.732	0.892	0.994	0.921	0.929	0.957	0.525	0.932
49	0.959	0.873	0.952	0.879	0.729	0.894	0.991	0.923	0.913	0.955	0.523	0.931
50	0.961	0.873	0.955	0.879	0.731	0.893	0.990	0.925	0.907	0.955	0.525	0.932
51	0.963	0.872	0.958	0.879	0.733	0.896	0.991	0.926	0.899	0.955	0.528	0.931
52	0.965	0.871	0.958	0.878	0.735	0.896	0.992	0.925	0.909	0.954	0.530	0.931
53	0.966	0.872	0.959	0.880	0.733	0.894	0.993	0.926	0.913	0.954	0.525	0.931

54	0.965	0.872	0.957	0.879	0.731	0.894	0.991	0.927	0.915	0.955	0.523	0.932
55	0.968	0.873	0.963	0.880	0.736	0.894	0.998	0.927	0.900	0.954	0.525	0.932
56	0.969	0.872	0.963	0.879	0.738	0.896	0.998	0.927	0.908	0.953	0.529	0.932
57	0.972	0.872	0.964	0.880	0.737	0.894	0.996	0.927	0.909	0.953	0.527	0.931
58	0.973	0.873	0.964	0.880	0.736	0.894	0.994	0.928	0.915	0.954	0.523	0.931
59	0.974	0.872	0.965	0.880	0.738	0.894	0.995	0.928	0.917	0.956	0.521	0.931
60	0.975	0.872	0.965	0.879	0.739	0.894	0.995	0.927	0.911	0.955	0.522	0.932
61	0.977	0.871	0.966	0.878	0.740	0.895	0.997	0.927	0.912	0.955	0.525	0.932
62	0.977	0.872	0.969	0.879	0.742	0.893	0.997	0.929	0.915	0.953	0.525	0.931
63	0.977	0.873	0.970	0.880	0.740	0.895	0.996	0.931	0.922	0.954	0.525	0.931
64	0.977	0.873	0.970	0.881	0.741	0.896	0.997	0.932	0.925	0.954	0.523	0.931
65	0.979	0.872	0.971	0.879	0.746	0.898	1.000	0.932	0.939	0.956	0.523	0.931
66	0.978	0.871	0.969	0.878	0.746	0.898	0.998	0.930	0.936	0.955	0.527	0.931
67	0.981	0.871	0.970	0.877	0.746	0.897	1.000	0.930	0.933	0.954	0.527	0.931
68	0.982	0.872	0.972	0.879	0.745	0.897	1.000	0.931	0.924	0.954	0.526	0.931
69	0.982	0.873	0.975	0.881	0.743	0.896	0.999	0.932	0.917	0.953	0.524	0.931
70	0.982	0.875	0.977	0.885	0.740	0.893	0.996	0.936	0.912	0.952	0.520	0.931
71	0.986	0.876	0.982	0.885	0.743	0.891	1.000	0.937	0.928	0.954	0.518	0.931
72	0.989	0.874	0.984	0.882	0.747	0.892	1.000	0.935	0.957	0.957	0.519	0.931

[Note: The column of temporal scale *s* lists the number of temporal units. One temporal unit represents 20 minutes. For example, the value of 3 represents a temporal scale of $3 \times 20 = 60$ minutes.]

Table S7| The comparison of allometric relations between population fluctuations and two different urban densities in terms of the ranges and averages of the goodness of fit, R^2, for the six focal cases.

City Name	$A(s)$ for population density		$A(s)$ for POI density	
	R^2	Avg. R^2	R^2	Avg. R^2
Beijing	0.886 – 0.897	0.890	0.871 – 0.893	0.878
Shanghai	0.943 – 0.958	0.948	0.876 – 0.899	0.884
Guangzhou	0.914 – 0.920	0.915	0.886 – 0.898	0.892
Shenzhen	0.946 – 0.966	0.956	0.905 – 0.937	0.920
Milan	0.787 – 0.814	0.797	0.912 – 0.957	0.946
Greater Boston	0.816 – 0.842	0.834	0.921 – 0.933	0.931

[Note: Avg. R^2 refers to the average of the goodness of fit, R^2.]

Supplementary Figures:

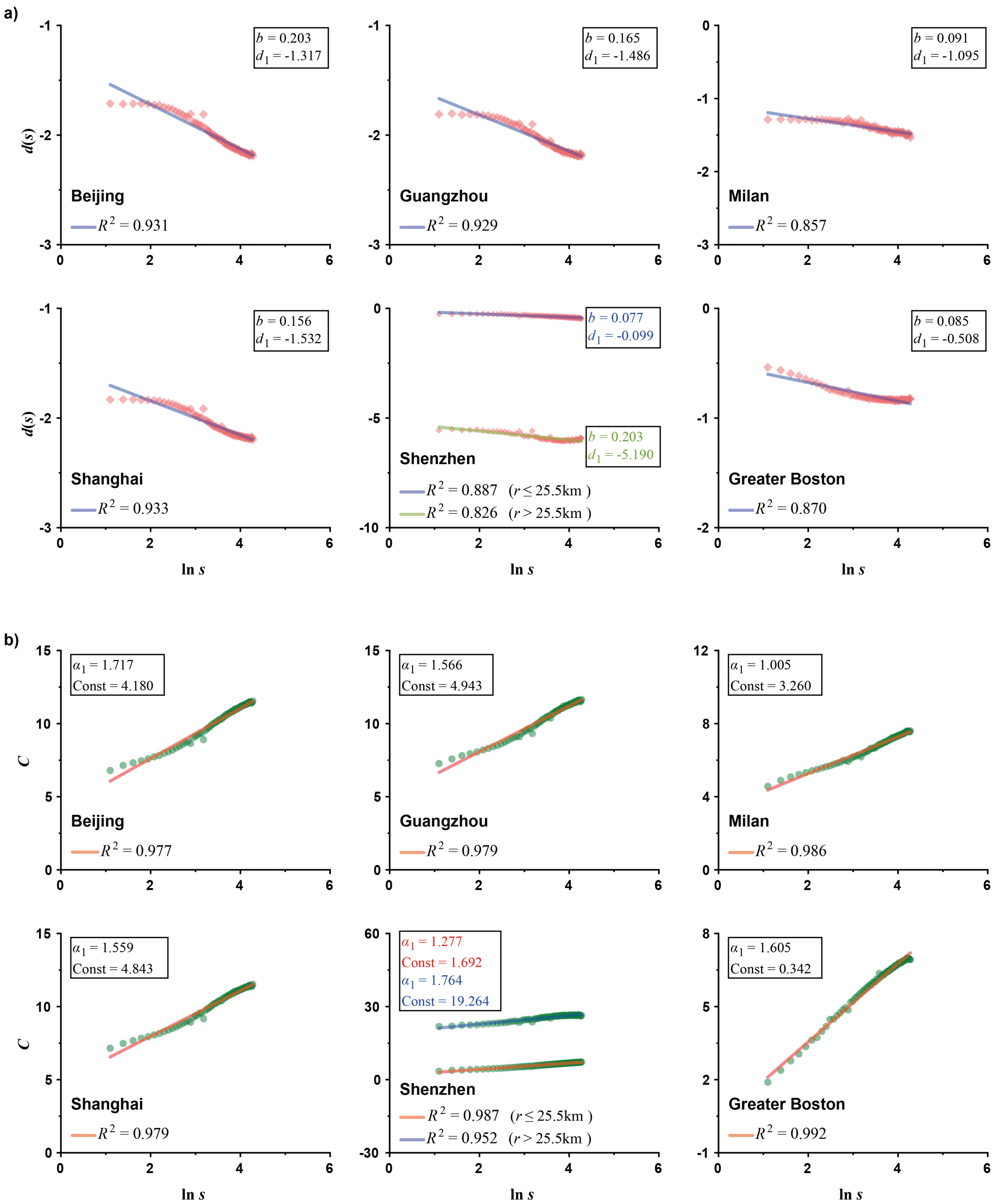

Fig. S19| Temporal gradients of the estimated spatial scaling exponents $d(s)$ and parameters C for population fluctuations. a, Plots of the estimated spatial scaling exponent of population fluctuations $d(s)$ and the logarithm of its corresponding temporal scale s for the six focal cases. The linearity in the plots indicates a temporally logarithmic gradient pattern of $d(s)$ as expressed in Eq. (5). The estimation of the slope coefficient and intercept yields the spatiotemporal gradient rate b and theoretical scaling exponent d_1 of population fluctuations in one temporal unit ($s = 1$) respectively. **b**, Plots of the estimated parameters C and the logarithm of its corresponding temporal scale s for

the six focal cases. The parameter C is defined as $C = \alpha_1 \ln s + \text{Const}$ in Eq. (13) based on the derived Eq. (12), suggesting the linearity in the plots. The estimation of the slope coefficient and intercept yields the theoretical scaling exponent α_1 near the city center ($r = 1$) and the constant respectively.

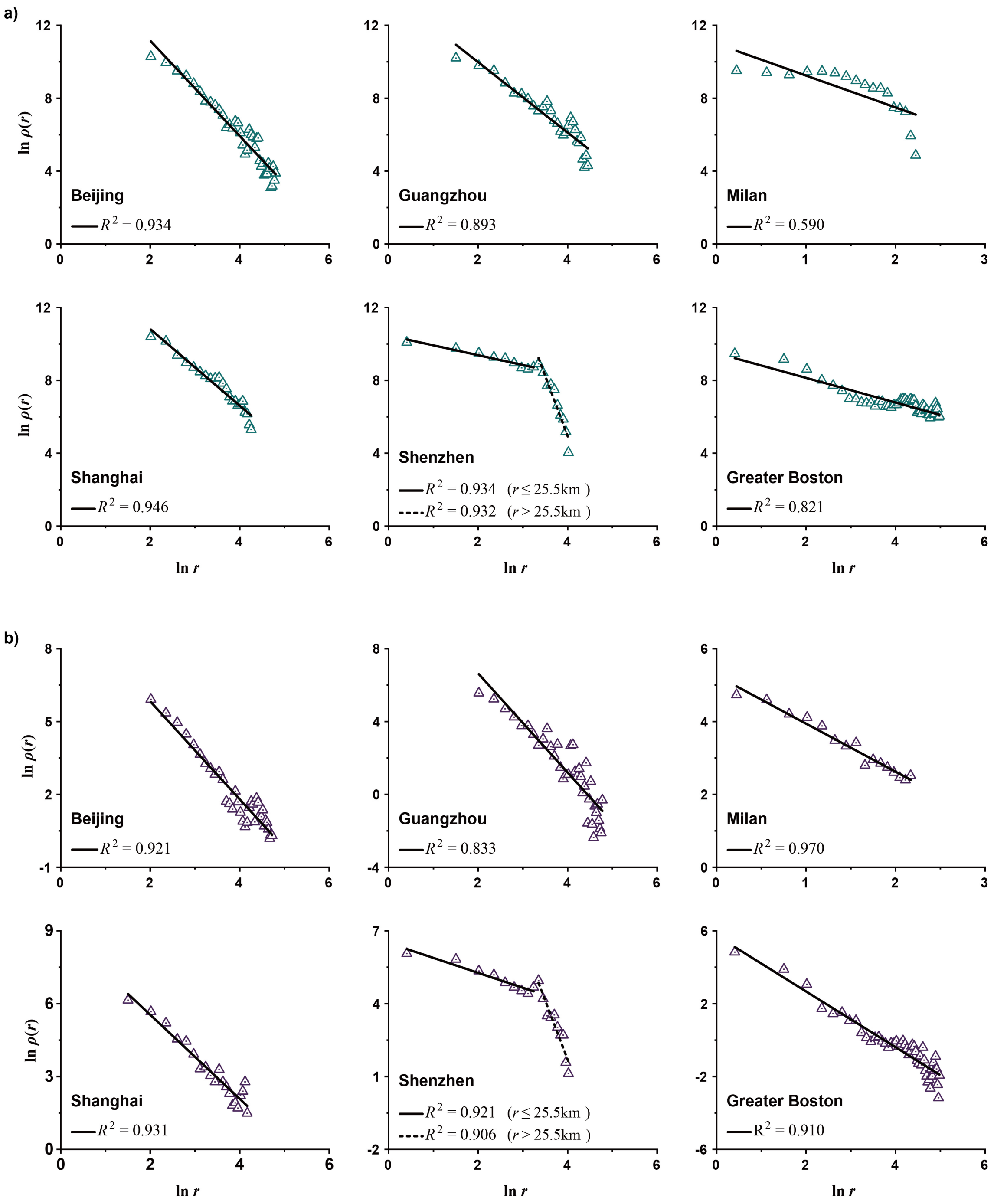

Fig. S20| Double-logarithm plots of urban density $\rho(r)$ (a, the population density and b, the POI density) and the corresponding distance r from the closest city center of the six focal cases. The two values (upper and lower) of the goodness of fit, R^2, in the plot of Shenzhen correspond to the two scaling ranges ($r \le 25.5$ km and $r > 25.5$ km) of the plot in Fig. 3b, respectively.

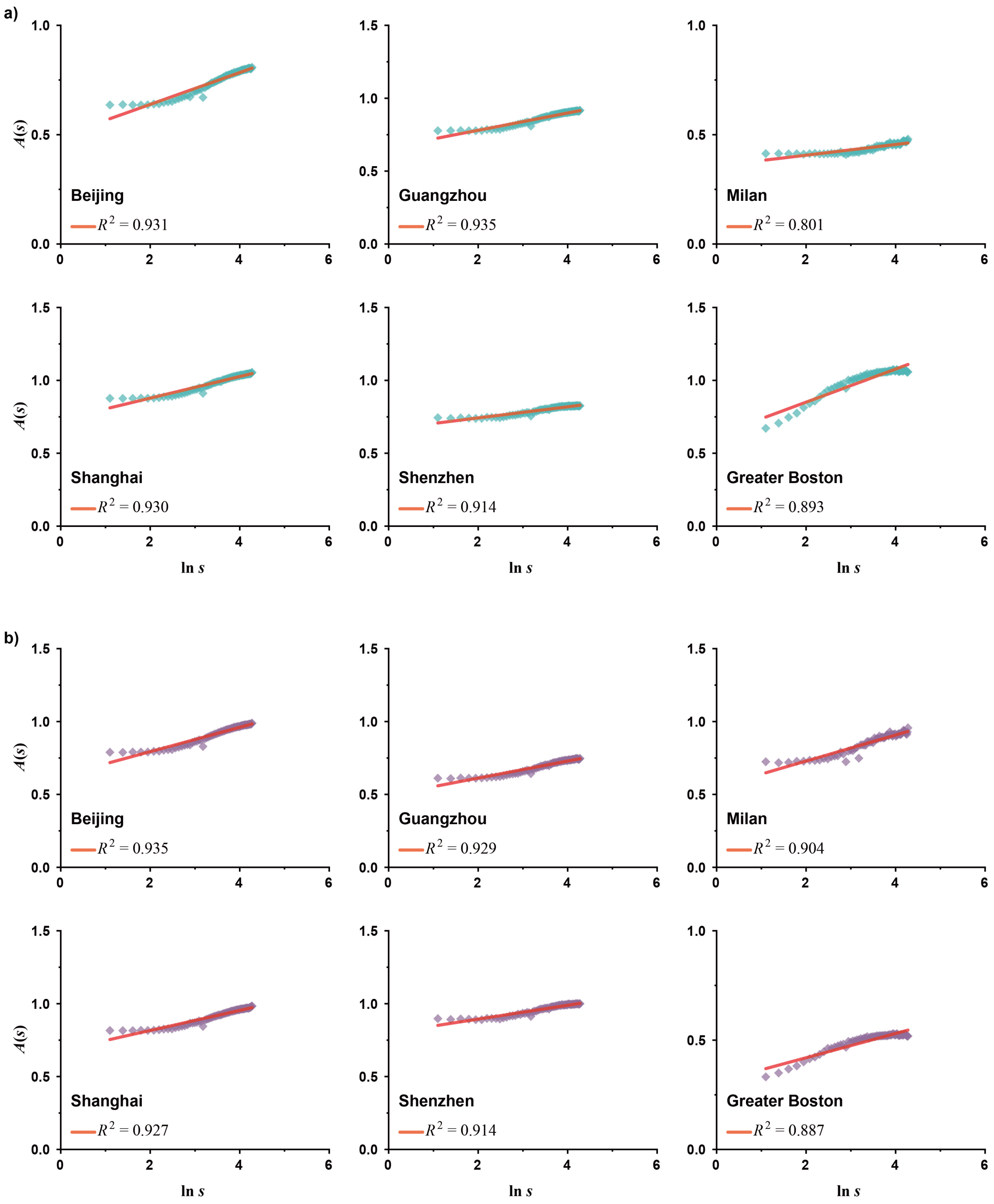

Fig. S21| Temporal gradient of the estimated allometric exponents $A(s)$. a, Plots of the allometric exponents $A(s)$ and the logarithm of its corresponding temporal scale s for the population densities of the six focal cases. **b**, Plots of the allometric exponents $A(s)$ and the logarithm of its corresponding temporal scale s for the POI densities of the six focal cases. The linearity in the plots indicates a logarithmic temporal gradient of the allometric exponents $A(s)$ as derived in Eq. (6).

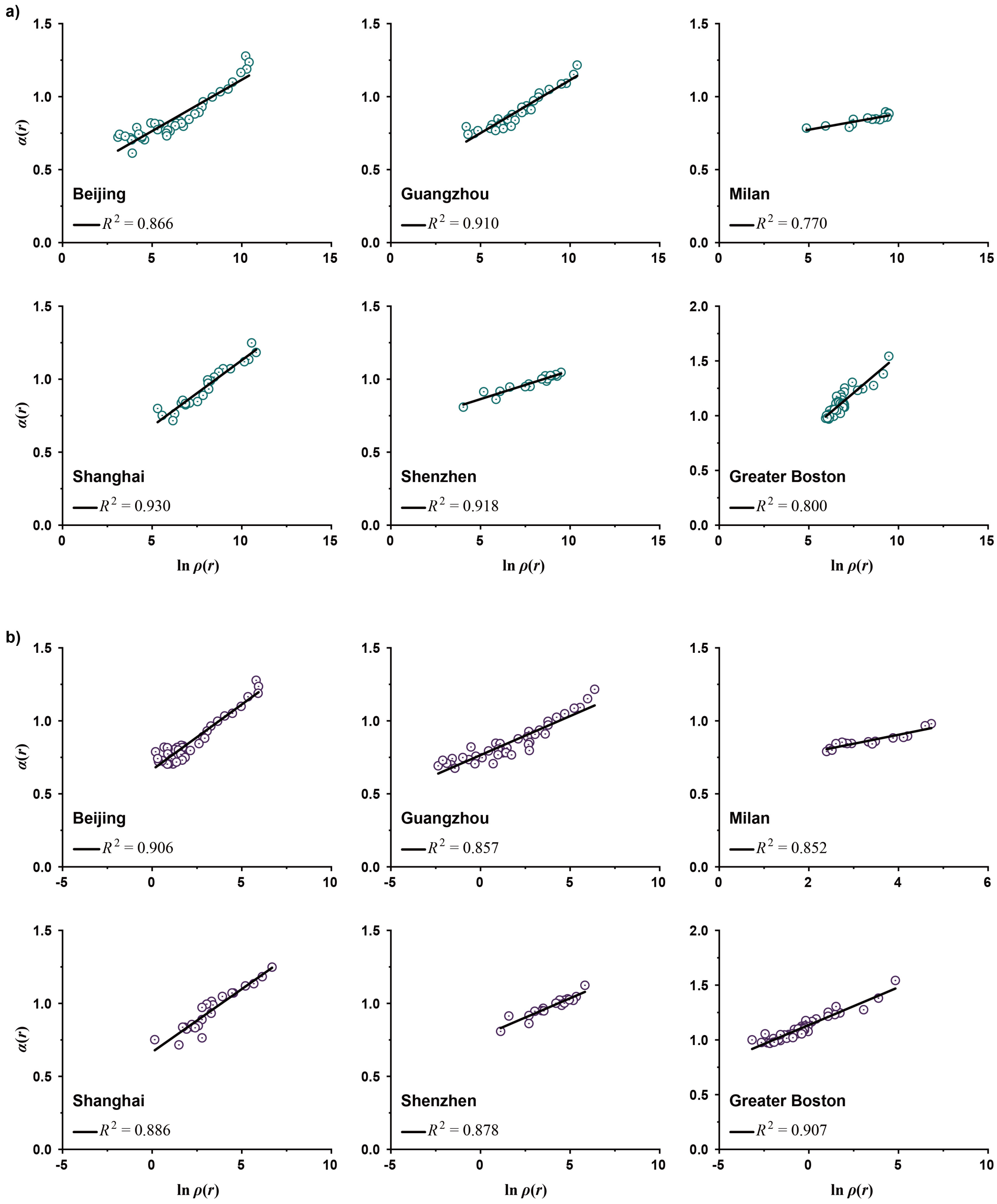

Fig. S22| Density gradient of the estimated temporal scaling exponents $\alpha(r)$. a, Plots of the fluctuation scaling exponent $\alpha(r)$ and the logarithm of its corresponding population density $\ln \rho(r)$ for the six focal cases. **b**, Plots of the fluctuation scaling exponent $\alpha(r)$ and the logarithm of its corresponding POI density $\ln \rho(r)$ for the six focal cases. The linearity in the plots indicates a logarithmic relationship between the DFA fluctuation exponent $\alpha(r)$ and population density $\rho(r)$ as derived from Eqs. (17) and (18).

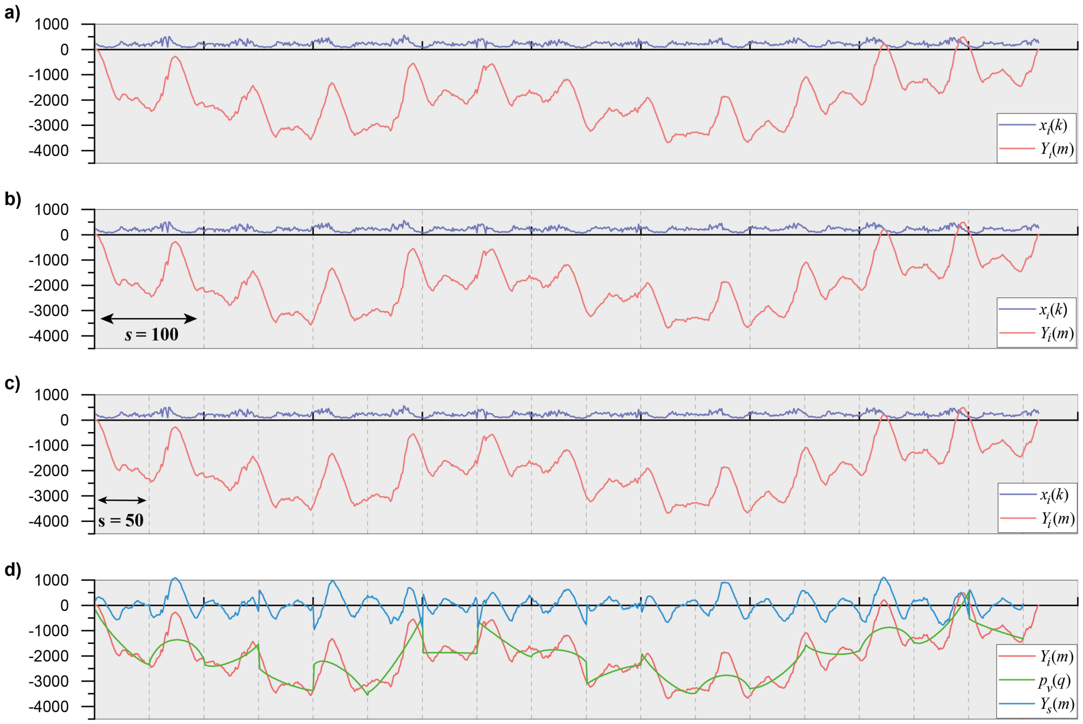

Fig. S23| An example of the process of temporally weighted detrended fluctuation analysis. a, A sketch map of global profile creation. **b**, An example of global profile divisions with the temporal scale $s = 100$. **c**, An example of global profile divisions with the temporal scale $s = 50$. **d**, An example of the local detrending process for $s = 50$. $x_i(k)$ represents the original time series. $Y_i(m)$ denotes the profile generated from $x_i(k)$ based on Eq. (7). $Y_s(m)$ refers to the detrended profile obtained by subtracting the local fits $p_v(q)$ from $Y_i(m)$.

References

1. Dong, L., Duarte. F., Duranton, G., Santi, P., Barthelemy, M., Batty, M., Bettencourt, L., Goodchild, M., Hack, G., Liu, Y., Pumain. D., Shi, J., Verbavatz, V., West, G. B., Yeh, Y., and Ratti, C. 2024. Defining a city: delineating urban areas using cell-phone data, *Nature Cities*, https://doi.org/10.1038/s44284-023-00019-z.
2. Defourny, P., Lamarche, C., Bontemps, S., De Maet, T., Van Bogaert, E., Moreau, I., Brockmann, C., Boettcher, M., Kirches, G., Wevers, J., Santoro, M., Ramoino, F., and Arino, O., 2017. Land Cover CCI Product User Guide Version 2.0.
3. Kantelhardt, J. W. 2009. Fractal and multifractal time series. In *Encyclopedia of Complexity and Applied Systems Science*, ed. Meyers, R. A., 3754-3778. New York: Springer.
4. Goldberger, A. L., Amaral, L. A. N., Hausdorff, J. M., Ivanov, P. C., Peng, C.-K., and Stanley, H. E. 2002. Fractal dynamics in physiology: Alterations with disease and aging. *Proceedings of the National Academy of Sciences*, *99*(Suppl 1), 2466–2472.
5. Weber, R. O., and Talkner, P. 2001. Spectra and correlations of climate data from days to decades. *Journal of Geophysical Research*, *106*(D17), 20131–20144.
6. Kantelhardt, J. W., Rybski, D., Zschiegner, S. A., Braun, P., Koscielny-Bunde, E., Livina, V., Havlin, S., & Bunde, A. 2003. Multifractality of river runoff and precipitation: comparison of fluctuation analysis and wavelet methods. *Physica A*, *330*(1), 240–245.
7. Mandelbrot, B. B., and Van Ness, J. W. 1968. Fractional Brownian Motions, Fractional Noises and Applications. *SIAM Review*, *10*(4), 422–437.
8. Mandelbrot, B. B. 1983. *The Fractal Geometry of Nature*. New York: Freeman.
9. Eke, A., Hermán, P., Bassingthwaighte, J. B., Raymond, G. M., Percival, D. B., Cannon, M., Balla, I., and Ikrényi, C. 2000. Physiological time series: Distinguishing fractal noises from motions. *Pflügers Archiv*, *439*(4), 403–415.
10. Eke, A., Herman, P., Kocsis, L., and Kozak, L. R. 2002. Fractal characterization of complexity in temporal physiological signals. *Physiological Measurement*, *23*(1), R1.
11. Alstro, P., Trunfio, P. A., and Stanley, H. E. 1990. Spatiotemporal fluctuations in growth phenomena: Dynamical phases and 1/f noise. *Physical Review A*, *41*(6), 3403.
12. Bak, P. 1996. *How Nature Works: The Science of Self-organized Criticality*. Springer New York.

13. Chen, Y., and Zhou, Y. 2008. Scaling laws and indications of self-organized criticality in urban systems. *Chaos, Solitons & Fractals*, *35*(1), 85-98.
14. Schaefer, A., Brach, J. S., Perera, S., and Sejdić, E. 2014. A comparative analysis of spectral exponent estimation techniques for 1/fβ processes with applications to the analysis of stride interval time series. *Journal of Neuroscience Methods*, *222*, 118–130.
15. Malamud, B. D., and Turcotte, D. L. 1999. Self-affine time series: measures of weak and strong persistence. *Journal of Statistical Planning and Inference*, *80*(1), 173–196.
16. Hurst, H. E. 1951. Long-term storage capacity of reservoirs. *Transactions of the American Society of Civil Engineers*, 116, 770-808.
17. Feder, J. 1988. *Fractals*. Plenum Press.
18. Delignieres, D. 2015. Correlation Properties of (Discrete) Fractional Gaussian Noise and Fractional Brownian Motion. *Mathematical Problems in Engineering*, *2015*, 1–7.
19. Bak, P., Chen, K., and Creutz, M. 1989. Self-organized criticality in the Game of Life. *Nature*, *342*(6251), 780–782.
20. Batty, M., and Xie, Y. 1999. Self-organized Criticality and Urban Development. *Discrete Dynamics in Nature and Society*, *1999*(2-3), 109–124.
21. Majumdar, S. N., and Dhar, D. 2001. Persistence in a stationary time series. *Physical Review. E, Statistical, Nonlinear, and Soft Matter Physics*, *64*(4), 046123.
22. Caccia, D. C., Percival, D., Cannon, M. J., Raymond, G., and Bassingthwaighte, J. B. 1997. Analyzing exact fractal time series: evaluating dispersional analysis and rescaled range methods. *Physica A*, *246*(3), 609–632.
23. Higuchi, T. 1988. Approach to an irregular time series on the basis of the fractal theory. *Physica D: Nonlinear Phenomena*, *31*(2), 277-283.
24. DePetrillo, P. B., and Ruttimann, U. E. 1999. Determining the Hurst exponent of fractal time series and its application to electrocardiographic analysis. *Computers in Biology and Medicine*, *29*(6), 393-406.
25. Zhou, Y., and Leung, Y. 2010. Multifractal temporally weighted detrended fluctuation analysis and its application in the analysis of scaling behavior in temperature series. *Journal of Statistical Mechanics: Theory and Experiment, 2010*(06), P06021.
