# Supplementary material for: The spatiotemporal scaling laws of urban population dynamics
Source: Nat Commun. 2025 Mar 24;16:2881. doi: 10.1038/s41467-025-58286-4 (PMC11933343; doi:10.1038/s41467-025-58286-4)
Supplement: Supplementary file 3 — Reporting Summary [file 41467_2025_58286_MOESM3_ESM.pdf]

nature portfolio

Corresponding author(s): Bo Huang

Last updated by author(s): Feb 13, 2025

Reporting Summary

Nature Portfolio wishes to improve the reproducibility of the work that we publish. This form provides structure for consistency and transparency in reporting. For further information on Nature Portfolio policies, see our Editorial Policies and the Editorial Policy Checklist.

Statistics

For all statistical analyses, confirm that the following items are present in the figure legend, table legend, main text, or Methods section.

n/a	Confirmed	
☐	☒	The exact sample size (*n*) for each experimental group/condition, given as a discrete number and unit of measurement
☐	☒	A statement on whether measurements were taken from distinct samples or whether the same sample was measured repeatedly
☒	☐	The statistical test(s) used AND whether they are one- or two-sided<br>*Only common tests should be described solely by name; describe more complex techniques in the Methods section.*
☒	☐	A description of all covariates tested
☐	☒	A description of any assumptions or corrections, such as tests of normality and adjustment for multiple comparisons
☐	☒	A full description of the statistical parameters including central tendency (e.g. means) or other basic estimates (e.g. regression coefficient) AND variation (e.g. standard deviation) or associated estimates of uncertainty (e.g. confidence intervals)
☒	☐	For null hypothesis testing, the test statistic (e.g. *F*, *t*, *r*) with confidence intervals, effect sizes, degrees of freedom and *P* value noted<br>*Give P values as exact values whenever suitable.*
☒	☐	For Bayesian analysis, information on the choice of priors and Markov chain Monte Carlo settings
☒	☐	For hierarchical and complex designs, identification of the appropriate level for tests and full reporting of outcomes
☒	☐	Estimates of effect sizes (e.g. Cohen's *d*, Pearson's *r*), indicating how they were calculated

Our web collection on statistics for biologists contains articles on many of the points above.

Software and code

Policy information about availability of computer code

Data collection	The data were collected from various websites through data providers' APIs using Python or used through commercial cooperation with their permission.
Data analysis	The code used for data analysis was custom developed in Python (3.6) and Matlab (R2022b). It can be found at GitHub (https://github.com/Xingyetan89/Data-processing-for-investigation-on-population-dynamics.git) and Zenodo (https://doi.org/10.5281/zenodo.14855190).

For manuscripts utilizing custom algorithms or software that are central to the research but not yet described in published literature, software must be made available to editors and reviewers. We strongly encourage code deposition in a community repository (e.g. GitHub). See the Nature Portfolio guidelines for submitting code & software for further information.

Data

Policy information about availability of data

All manuscripts must include a data availability statement. This statement should provide the following information, where applicable:

- Accession codes, unique identifiers, or web links for publicly available datasets
- A description of any restrictions on data availability
- For clinical datasets or third party data, please ensure that the statement adheres to our policy

The mobile device data from Tencent and SafeGraph in this study are not publicly available due to licensing concern and privacy protection.

The preprocessed time series data can only be available with the permission from the data provider.

The mobile phone data for Milan were released under the Open Database License (1.0) and are freely available at https://doi.org/10.7910/DVN/EGZHFV.

Population data were derived from WorldPop [https://www.worldpop.org/datacatalog/], which provides open spatial demographic data.

The data of points of interest for Chinese cities were crawled from Amap [https://www.amap.com/], while that for for Milan and Greater Boston were downloaded from OpenStreetMap [https://www.openstreetmap.org/] that provides open map data. Due to licensing concerns, the crawled data are not publicly available.

Research involving human participants, their data, or biological material

Policy information about studies with human participants or human data. See also policy information about sex, gender (identity/presentation), and sexual orientation and race, ethnicity and racism.

Reporting on sex and gender	N/A
Reporting on race, ethnicity, or other socially relevant groupings	N/A
Population characteristics	N/A
Recruitment	N/A
Ethics oversight	N/A

Note that full information on the approval of the study protocol must also be provided in the manuscript.

Field-specific reporting

Please select the one below that is the best fit for your research. If you are not sure, read the appropriate sections before making your selection.

☐ Life sciences ☒ Behavioural & social sciences ☐ Ecological, evolutionary & environmental sciences

For a reference copy of the document with all sections, see nature.com/documents/nr-reporting-summary-flat.pdf

Behavioural & social sciences study design

All studies must disclose on these points even when the disclosure is negative.

Study description	This study was conducted using quantitative methods. We discovered an undocumented spatiotemporal scaling law that governs the patterns and regularities of urban population dynamics over space and time, as well as a new urban allometry linking population dynamics with density and functional attractiveness. We validated these discoveries with six case studies (i.e., Beijing, Shanghai, Guangzhou, Shenzhen, Milan, and Greater Boston).
Research sample	The check-in data of Chinese cities were randomly collected from Tencent users, covering more than 70% of the population in China. The mobile phone data for Milan were obtained from Telecom Italia users, representing up to 34% of the population due to market share limitations. Additionally, SafeGraph data were utilized, with check-in data covering approximately 15% of the population in the selected cities. The selection of these study cases was primarily based on the availability of mobile device data. Despite this, these cases exhibit apparent diversity in geographical location, urban development history, culture, spatial range, population size, function and status, urban centrality, and spatial structure. Such diversity renders these cases representative in testing our results.
Sampling strategy	There is no sampling performed in this study. The sample size is determined by the size of original datasets.
Data collection	The check-in records of Chinese cities were crawled from Tencent's Xingyun map in 2018 (April 16 ~ 27, 2018) when the data can be publicly accessed through the website front-end API with no permission restrictions. The mobile phone data for Milan released under the Open Databased License (1.0) were directly downloaded on the date of November 15, 2022. The GPS data of Greater Boston were provided by SafeGraph for research usage on May 27, 2024.<br><br>Population density data are openly available on WorldPop and were directly downloaded on October 21, 2022 (Chinese cities), November 21, 2022 (Milan), and June 3, 2025 (Greater Boston).<br><br>The POI data of Milan and Greater Boston were directly downloaded from OpenStreetMap on November 19, 2023 and May 29, 2025, respectively, and that of Chinese cities were crawled from Amap on March 5, 2018.
Timing	The check-in records of Chinese cities from Tencent were obtained in 2018 (April 16 ~ 27, 2018). The mobile phone data for Milan refer to the year 2013 (November 04 ~ 15, 2013). GPS pings data of Greater Boston from SafeGraph refer to the year 2020 (February 03 ~ 14, 2020). The years of population and POI data align with that of mobile device data for all the study cases.
Data exclusions	No data were excluded from the analyses.

Non-participation	N/A
Randomization	N/A

Reporting for specific materials, systems and methods

We require information from authors about some types of materials, experimental systems and methods used in many studies. Here, indicate whether each material, system or method listed is relevant to your study. If you are not sure if a list item applies to your research, read the appropriate section before selecting a response.

Materials & experimental systems

n/a	Involved in the study
☒	☐ Antibodies
☒	☐ Eukaryotic cell lines
☒	☐ Palaeontology and archaeology
☒	☐ Animals and other organisms
☒	☐ Clinical data
☒	☐ Dual use research of concern
☒	☐ Plants

Methods

n/a	Involved in the study
☒	☐ ChIP-seq
☒	☐ Flow cytometry
☒	☐ MRI-based neuroimaging

Plants

Seed stocks	N/A
Novel plant genotypes	N/A
Authentication	N/A
